# The hexokinase “HKDC1” interaction with the mitochondria is essential for liver cancer progression

**DOI:** 10.1038/s41419-022-04999-z

**Published:** 2022-07-28

**Authors:** Md. Wasim Khan, Alexander R. Terry, Medha Priyadarshini, Vladimir Ilievski, Zeenat Farooq, Grace Guzman, Jose Cordoba-Chacon, Issam Ben-Sahra, Barton Wicksteed, Brian T. Layden

**Affiliations:** 1 Division of Endocrinology, Diabetes and Metabolism, Department of Medicine, University of Illinois at Chicago, Chicago, IL 60612 USA; 2 Department of Biochemistry and Molecular Genetics, College of Medicine, University of Illinois at Chicago, Chicago, IL 60607 USA; 3 Department of Pathology, College of Medicine, Cancer Center, University of Illinois Hospital and Health Science Chicago, Chicago, IL 60612 USA; 4 Department of Biochemistry and Molecular Genetics, Feinberg School of Medicine, Northwestern University, Chicago, IL 60611 USA; 5 Jesse Brown Veterans Affairs Medical Center, Chicago, IL 60612 USA

**Keywords:** Liver cancer, Kinases

## Abstract

Liver cancer (LC) is the fourth leading cause of death from cancer malignancies. Recently, a putative fifth hexokinase, hexokinase domain containing 1 (HKDC1), was shown to have significant overexpression in LC compared to healthy liver tissue. Using a combination of in vitro and in vivo tools, we examined the role of HKDC1 in LC development and progression. Importantly, HKDC1 ablation stops LC development and progression via its action at the mitochondria by promoting metabolic reprogramming and a shift of glucose flux away from the TCA cycle. HKDC1 ablation leads to mitochondrial dysfunction resulting in less cellular energy, which cannot be compensated by enhanced glucose uptake. Moreover, we show that the interaction of HKDC1 with the mitochondria is essential for its role in LC progression, and without this interaction, mitochondrial dysfunction occurs. As HKDC1 is highly expressed in LC cells, but only to a minimal degree in hepatocytes under normal conditions, targeting HKDC1, specifically its interaction with the mitochondria, may represent a highly selective approach to target cancer cells in LC.

## Abstract (web summary)

Mitochondrial interaction of HKDC1 is essential for its pro-proliferative role in LC. When HKDC1 is ablated in liver cancer cells, mitochondrial dysfunction occurs which results in decrease ATP in the cells. The cancer cells react to decreased cellular energy by increasing glucose uptake. Also due to mitochondrial dysfunction there is enhanced ER stress and ER-mitochondrial interaction, leading to more calcium flux into the mitochondria. This multi-pronged effects of HKDC1 ablation leads to decreased cellular proliferation in LC.

## Graphical abstract

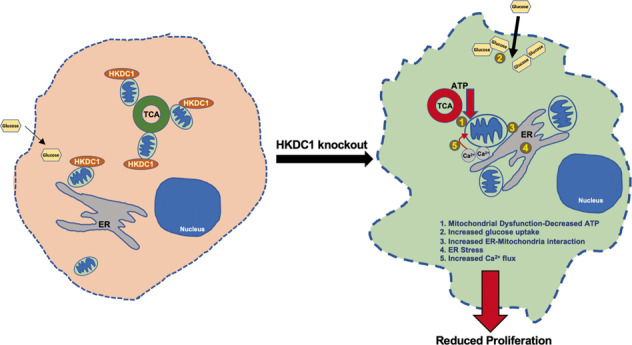

## Introduction

Globally, there has been a decline in the number of deaths due to cancer malignancies; however, the incidence and mortality due to LC continues to rise [1] and is now the fourth leading cause of cancer death worldwide [2–4]. Currently, a cure for LC is feasible only if the disease is localized and amenable to complete surgical resection. Since the risk of LC recurrence is high, there is a need to identify novel targets for prognostic and diagnostic opportunities [5]. One possible target is the phenomenon of “metabolic reprogramming” where cancer cells have an over-dependency on glucose with dysregulated glucose metabolism that can be therapeutically targeted [6, 7].

Hexokinases (HKs) catalyze the first step in glucose metabolism and thus contributes to the rate of cell growth and proliferation [8, 9]. While HK1 is widely and constitutively expressed, HK2 is selectively expressed during embryogenesis and in cancer where it has been shown to induce proliferation and inhibit cell death [8–12]. A major driver in the development of LC is hepatocyte mitochondrial dysfunction by various possible mechanisms, which augments LC progression such as (i) generation of ROS, (ii) changes in activities of mitochondrial enzymes involved in TCA cycle leading to accumulation of oncometabolites that aid in proliferation and, (iii) defects in the mitochondria, which may lead to faulty apoptotic pathways [13–16]. HK1 and HK2 have been shown to interact with the mitochondria and contribute to LC progression through this interaction [13, 17–19]. We recently discovered a novel fifth HK, hexokinase domain containing 1 (HKDC1), which has been shown to be overexpressed in certain cancers compared to healthy tissue, and most significantly in LC, where it interacts with the mitochondria [20–27]. These data suggest that HKDC1 plays a pivotal role in LC progression, possibly via its action at the mitochondria. In this context, we hypothesized that the novel hexokinase, HKDC1, by interacting with the mitochondria plays previously unidentified roles in LC progression.

## Materials and methods

### Animal studies

All animal experiments were approved by the University of Illinois at Chicago Institutional Animal Care and Use Committee, as required by United States Animal Welfare Act, and the NIH’s policy.

**Fig. 1:**
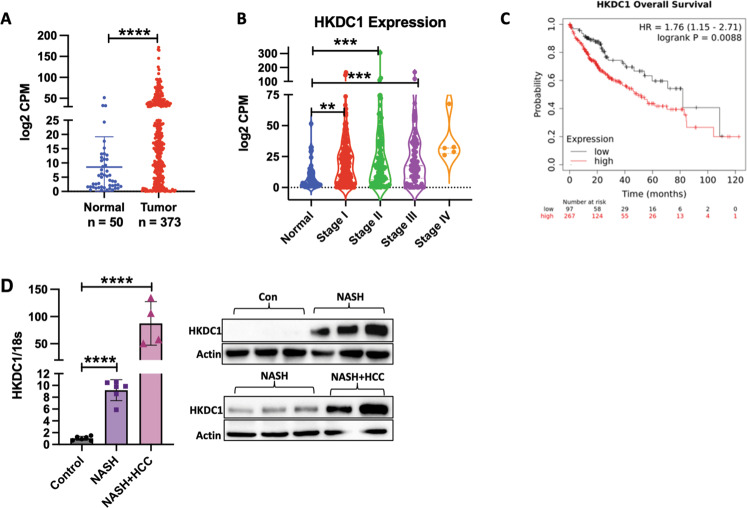
HKDC1 is upregulated in LC. mRNA expression data of HKDC1 in liver cancer (LIHC) in human patients mined from the TCGA dataset is shown for **A** normal and LC patient samples where T denotes tumor (*n* = 369) and *N* = denotes normal surrounding tissue (*n* = 50) and **B** to show HKDC1 expression in samples from different tumor stages (stage I (*n* = 173); stage II (*n* = 87); stage III (*n* = 85); stage IV (*n* = 5); compared to normal tissue (*n* = 50). **C** Kaplan–Meier survival curves were plotted from TCGA dataset using the website (https://kmplot.com/analysis/) with no restrictions applied; HR = hazard ratio. **D** HKDC1 mRNA (left panel) and protein expression (right panel) from liver (non-tumor regions) of mice fed control diet (Con) or NASH diet (NASH) over 20 weeks (*n* = 5 for control and NASH; *n* = 3 for NASH + LC), mice that developed LC along with NASH are denoted NASH + LC. Values are mean ± SD; *p < 0.05; **p < 0.01; ***p < 0.001; ****p < 0.0001 by student’s *t*-test (for **A**) or one-way ANOVA (for **B**, **D**).

For Fig. 1D, mice were either fed low fat, cholesterol, and fructose (LFCF, Control, Cat # D09100304) or the high fat, cholesterol, and fructose (HFCF, NASH diet, Cat # D16010101, Research diets, Inc) diets for 24 weeks.

#### DEN model

For diethylnitrosamine (DEN)-induced hepatocarcinogenesis, HKDC1 floxed mice were crossed with Albumin-Cre mice (Jackson Lab, Stock No: 003574) [28] to create a liver-specific HKDC1 knockout mouse model (HKDC1-LKO). DEN-induced hepatocarcinogenesis was performed by intraperitoneal injection of (25 mg/kg body weight) in 14 days old male mice [29]. Livers from 10-month-old treated mice were analyzed macroscopically for tumor lesions. Livers were then collected fixed in 10% formalin for 18 h and subsequently preserved with 70% ethanol. Fixed tissues were then processed and embedded in paraffin. Paraffin embedded tissues were processed and 5 μM slides were prepared for BrdU and Ki67 staining.

#### Xenograft models

HepG2 cells were deleted for HKDC1 by CRISPR/Cas9 (described below). Male athymic nude mice (5 weeks old) were subcutaneously injected into the right flank with 1 × 10^6^ (in 100 μL) HepG2 cells-expressing either empty vector (EV) or HKDC1-KO cells. When tumors were palpable (~100 mm^3^), tumor size was monitored twice per week and measurements were taken with a Vernier caliper till 16 weeks post implantation. Tumor volume was calculated by the formula (*a* × *b*^2^)/2, where *a* and *b* are length and breadth, respectively. End points were reached, and mice were sacrificed once the tumor size measured 2 cm.

For the inducible shRNA-mediated HKDC1 knockdown experiments, modulated Hep3B2 cells were grown in culture before collection for injection subcutaneously into the right flanks of each mouse (1 × 10^6^ in 100 μL per site). Once tumors were ~65 mm^3^ in volume, mice from each group were given a treatment with a (200 mg/kg) doxycycline diet (Bioserve) for 7 days. Tumor volume was calculated by the formula (*a* × *b*^2^)/2 where a and b are length and breadth, respectively. End points were reached, and mice were sacrificed once the tumor size measured 2 cm.

### ATP assay

Total ATP production was measured through an ATP Assay Kit (Abcam, ab83355). In brief, cells were harvested, washed with PBS, and then resuspended in 100 μL of ATP assay buffer. Cells were homogenized and then centrifuged (4 °C at 13,000 × *g*) to remove any insoluble material. The supernatants were then collected and deproteinization was done using Abcam Sample preparation kit (ab204708). Deproteinized samples were incubated with the ATP probe as per manufacturer’s recommendations. Absorbance was detected at 570 nm using a microplate reader (BioTek Instruments, Inc.). The results are presented as fold-change.

### BrdU assay

BrdU Cell Proliferation ELISA Kit (colorimetric; ab126556) was purchased from Abcam (UK) for BrdU assay analysis. Cells (3 × 10^4^) were added to each 96-well plate. Four hours after seeding, 20 μL BrdU was added to each well and incubated for 16 h. After incubation, the cells were fixed using a 200 μL/well fixing solution for 30 min. The cells removed from the fixing solution were later incubated with 100 μL/well anti-BrdU antibody for 1 h at room temperature. Next, 100 μL/well Peroxidase Goat anti-mouse IgG was added to the cells and then incubated for 30 min at room temperature. Finally, the absorbance was measured at 450 nm to assess the ability of the cells to proliferate. The net absorbance values of samples were obtained by subtracting the blank absorbance readings.

### Cell culture and reagents

HepG2 (ATCC HB-8065) and Huh7 cells were grown in Eagle’s Minimum Essential Medium (EMEM; ATCC; 30-2003), Hep3B2 cells were grown in Dulbecco’s Modified Eagle′s Medium-high glucose (Sigma, D642), SNU-475 (CRL-2236), cells were maintained in Roswell Park Memorial Institute Medium (RPMI; ATCC; 30-2001) and AML-12 cells were maintained in Dulbecco’s modified Eagle medium (DMEM)-F12 medium (ATCC; 30-2006). All growth media were supplemented with 10% fetal bovine serum (FBS; Sigma; F0926) and 1% penicillin–streptomycin (Gibco; 15070063). Cells were never allowed to grow beyond 70–80% confluency. All chemicals were from Sigma-Aldrich (St. Louis, MO, US) unless stated otherwise.

### Cell-cycle assay

Briefly, this assay was performed using 6-well plates with a concentration of 1 × 10^4^ cells per well. Further, the cells were trypsinized, washed with PBS, and fixed in cold 70% ethanol at −20 °C overnight. Thereafter, ethanol was removed, and the cells were resuspended in 500 mL of PBS containing 100 μg/mL of RNase, stained with 1 mg/mL of propidium iodide (Cat. No. P3566, Invitrogen, USA) for 30 min at RT. Further, based on the DNA fluorescence intensity, the percentage of cells in the G0/G1, S, and G2/M phases was determined by flow cytometric analysis using the FacsDIVA software (Becton-Dickinson Franklin Lakes, NJ, USA).

### Cell fractionation

Cytosolic, mitochondrial and nuclear fractions were separated by following the method of Holden and Horton. Briefly, cells at ~80–90% confluency harvested by adding 1× trypsin and washed two times with ice-cold 1× PBS. The pellet was resuspended in 400 μL of ice-cold buffer 1 (150 mM NaCl, 50 mM HEPES (pH 7.4), 25 μg/mL digitonin, protease inhibitor cocktail). This was further incubated at 4 °C in end-over-end rotation for 10 min, following which it was centrifuged at 2000 x *g* to pellet the cells. The supernatant was aspirated to obtain the cytosolic fraction. The cells were further washed with ice-cold 1× PBS and centrifuged at 100 x *g* at 4 °C to remove any digitonin. The pellet was resuspended by vortexing in 400 μL of ice-cold buffer 2 (150 mM NaCl, 50 mM HEPES, pH 7.4, 1% NP40, protease inhibitor) and incubated on ice for 30 min, following which it was centrifuged at 7000 x *g* to pellet down the nuclei and cellular debris. The supernatant, which comprised the mitochondrial fraction, was then aspirated. The pellet was further resuspended in 400 μL of ice-cold buffer 3 (150 mM NaCl, 50 mM HEPES, 0.1% SDS, pH 7.4, with protease and phosphatase inhibitor cocktail) and vortexed intermittently for 30 min. The suspension was incubated at 4 °C in end-over-end rotation for 1 h and centrifuged at 7000 x *g* for 10 min at 4 °C. The supernatant, which contained the nuclei, was collected by aspiration.

### Cell invasion assay

Cell invasion analysis was performed using a 24-well trans-well chamber (Cell invasion assay kit, ECM550, Sigma). Three-hundred microliters of warm serum-free medium was added to the upper inserts with an 8 μm pore size polycarbonate membrane precoated with ECMatrix and allowed this to rehydrate the ECMatrix layer for 1–2 h at room temperature and then carefully removed medium from the inserts without disturbing the membrane. HepG2 cells from different groups were incubated at a concentration of 2 × 10^5^ cells/mL in EMEM medium with serum free and were cultured in the upper inserts for 72 h at 37 °C in a CO_2_ incubator. Cells were allowed to invade towards medium containing 10% FBS in the bottom chamber. The non-invading cells on the upper membrane surface were removed with a cotton tip and the invasion cells attached to the lower membrane surface were stained with 0.1% crystal violet for 20 min at room temperature. Stained cells were dissolved in 10% acetic acid (200 μL/well) and 150 μL of the dye/solute mixture were transferred to a 96-well plate for colorimetric reading of absorbance with an enzyme-linked immunosorbent assay reader using 570 nm as test wavelength.

### Cell lysis and immunoblotting

Cells were rinsed with ice-cold PBS before lysis in buffer containing 50 mM Tris-HCl (pH 7.4), 100 mM NaCl, 1 mM EDTA, 1 mM EGTA and 1% Triton-X-100 with protease/phosphatase inhibitor (Roche) mixture. The soluble fractions of cell lysates were isolated by centrifugation at 12,000 × *g* for 15 min at 4 °C. Protein concentration was determined using Bradford reagent (Bio-Rad Laboratories, Hercules, CA, USA). In all, 30 μg of denatured proteins were separated by sodium dodecyl-sulfate polyacrylamide gel electrophoresis (Mini-PROTEAN TGX Gels 10%, Bio-Rad Laboratories) and transferred to 0.45 μm nitrocellulose membranes. Membranes were blocked with 5% nonfat, dried milk in Tris-buffered saline with 0.1% Tween-20 for 1 h at 25 °C, then incubated with primary antibodies overnight at 4 °C washed and incubated with secondary antibodies for 1 h at 25 °C. After washing, Clarity Western ECL Substrate (Bio-Rad) was added, and the light signal was detected and analyzed using a ChemiDoc MP and Image Lab Ver 6.0 (Bio-Rad). List of all antibodies used with dilutions is included in Supplementary Table 2.

### Cell proliferation assay

Proliferation rates were assessed in cell lines by proliferation curve analysis. Cells were plated on 96-cm clear bottom black well. For proliferation curves, 1 day of growth was allowed before the first cell count, and thereafter, counts were performed every other day for 3–4 total counts. Growth medium was replaced every day to ensure no nutrient depletion takes place. Total viable cell numbers of individual wells were determined by staining the cells with Hoechst and propidium iodide (PI) and counting on the Celigo Imaging cytometer (Nexcelom Bioscience LLC, MA, USA). Cells stained with PI were counted as dead and subtracted from total cells to get viable cell number. Cell numbers for each time point were averaged and plotted by scatter plot with standard deviation.

### Colony formation

Two-hundred cells were plated onto six-well culture plates and allowed to grow for 21 days with media changed every 3 days. Colonies were fixed with glutaraldehyde (6.0% v/v), stained with crystal violet (0.5% w/v) and imaged. Colonies were counted with the Image J software.

### Co-immunoprecipitation

In all, 1 × 10^6^ cells were grown in T25 cell culture flasks, when cells were 80% confluent, they were lysed using the CHAPS-lysis buffer (Cell Signalling #9852). Protein was collected by centrifuging the cell lysates at 12,000 x *g*. Protein homogenates were then incubated with primary antibody (1:200) overnight in cold room. Antibody-protein complexes were separated by Pure-Proteome magnetic A/G beads (Sigma LSKMAGAG02) using manufacturer’s protocol and run on an immunoblot to detect interacting partners.

### Crispr-Cas9-mediated HKDC1 knockout

HKDC1 sgRNA CRISPR All-in-One Lentivirus (Human) (Cat No. 234181110603) was purchased from ABM and HepG2 cells (70% confluent) were infected at an MOI of 50 (virus titer >1 × 10^7^ IU/mL). Cells were allowed to grow for 2 days post infection after which they were selected with puromycin (1 μg/mL) for 7 days. Selected cells were then used to perform a single cell colony formation in a 96-well plate. When colonies were formed (roughly 21 days), the cells from each well were subcultured for three generations more. HKDC1 knockout was confirmed in these clones by qPCR and immunoblot and then three individual clones were pooled together. Genome edit was confirmed by genomic sequencing in this pool and further HKDC1 knockout was confirmed by qPCR and western blotting.

### Glucose consumption

In all, 50 μL media was collected from cells growing in six-well plates at 70% confluency at 0, 2, 4, 6, and 16 h and stored at −80 °C. Glucose was measured in these aliquots with the Biosen R-Line lactate and glucose analyzer (EKF Diagnostics, TX, USA). To calculate glucose consumption, glucose remaining in the media at the indicated time-points was subtracted from the original glucose concentration at 0 h.

### Glucose uptake

Glucose uptake experiments were performed using 2-NBDG (2-(N-(7-nitrobenz-2-oxa-1,3-diazol-4-yl)amino)-2-deoxyglucose) (Invitrogen, Carlsbad, CA, USA) according to the manufacturer’s protocol. Briefly, cells were plated in a 96-well black clear bottom plate (Brand, Wertheim, Germany). After treatment, cells were washed three times with 1× PBS at room temperature and incubated for 30 min in zero glucose DMEM containing 75 μM 2-NBDG. Cells were then washed with ice-cold PBS three times. To each well, 200 μL of PBS was added and the relative fluorescence was measured in a fluorimeter (Synergy H1 multimode microplate reader; Biotek (Winooski, VT, USA); excitation 485 nm, emission 535 nm). The assay was normalized to total cellular protein.

### Hexokinase activity

Cells were grown in six-well plates with media change every 24 h. When cells reached 80% confluency, hexokinase activity was assayed in cell homogenates using the Hexokinase activity kit (MAK091, Sigma, St. Louis, MO, USA) according to the manufacturer’s instructions. Activity was normalized to the amount of protein in the sample.

### Immunohistochemistry

In all, 5 μm thick liver sections were first de-paraffinized with xylene and rehydrated through a series of decreasing percentages of ethanol. Antigen retrieval was performed by microwave heating in 1 mM ethylenediaminetetraacetic acid (EDTA), pH 8.0 or in 10 mM citrate buffer pH 6.0 for 5 min repeated four times. Cell and nuclear membrane permeabilization was performed by incubating sections in 0.25% Tween-20 in PBS for 30 min. Tissue sections were incubated with: rabbit polyclonal antibody to HKDC1 and mouse polyclonal antibody to VDAC at 1:100 dilution overnight at 4 °C. Appropriate secondary antibody donkey anti-rabbit antibody conjugated with Alexa Fluor 594 and donkey anti-mouse antibody conjugated with Alexa Fluor 647 were diluted at 1:800 for 1 h at room temperature. Nuclei were stained with ProLong Gold Antifade Mountant with DAPI (P36931, Thermo Fisher Scientific, Waltham, MA).

### Mitochondrial complex assays

Cells were grown in six-well plates with media change every 24–48 h. When cells reached 80% confluency, activities for mitochondrial Complex I, SDH and Complex III were performed with commercially available kits (Catalog # K968, K660 and K520; Biovision, CA, US) according to the manufacturer’s protocols.

### Metabolite measurements and analysis

For steady-state metabolomics, cells were grown in a 10 cm plate with media change every day. On the day of the experiment, cells were washed twice with PBS at room temperature and incubated with normal growth media for 2 h. For isotopic labeling analysis, cells were washed twice with warm PBS and incubated in medium containing 5.5 mm of [U-^13^C] glucose, 2 mM pyruvate and 2 mM glutamine for 4 h. After incubation, cells were washed twice with ice-cold saline and metabolites were extracted with 1 mL of ice-cold 80% methanol per plate. Cells were scraped and samples were frozen in liquid nitrogen and thawed in a 37 °C water bath three times. Following this, cells were centrifuged at 20,000 × *g* for 15 min at 4 °C. The supernatant was transferred to fresh tubes and dried. Samples were resuspended in 10 µL per 150,000 cells. Samples were analyzed by high-performance liquid chromatography and high-resolution mass spectrometry, and tandem mass spectrometry (HPLC-MS/MS). Specifically, the system consisted of a Thermo Q-Exactive in line with an electrospray source and an Ultimate3000 (Thermo) series HPLC consisting of a binary pump, degasser and auto-sampler outfitted with an Xbridge amide column (Waters; dimensions 4.6 × 100 mm and a 3.5-µm particle size). The mobile phase A contained 95% (v/v) water, 5% (v/v) acetonitrile, 20 mM ammonium hydroxide, 20 mM ammonium acetate, pH 9.0; B was 100% acetonitrile. The gradient was as follows: 0–1 min, 15% A; 18.5 min, 76% A; 18.5–20.4 min, 24% A; 20.4–20.5 min, 15% A; 20.5–28 min, 15% A with a flow rate of 400 μL/min. The capillary of the ESI source was set to 275 °C, with sheath gas at 45 arbitrary units, auxiliary gas at 5 arbitrary units and the spray voltage at 4.0 kV. In positive/negative polarity switching mode, an *m*/*z* scan range from 70 to 850 was chosen and MS1 data was collected at a resolution of 70,000. The automatic gain control (AGC) target was set at 1 × 10^6^ and the maximum injection time was 200 ms. The top 5 precursor ions were subsequently fragmented, in a data-dependent manner, using the higher energy collisional dissociation cell set to 30% normalized collision energy in MS2 at a resolution power of 17,500. Sample volumes of 150,000 cells in 10 μL were injected. Data acquisition and analysis were carried out by Xcalibur 4.0 software and Tracefinder 2.1 software, respectively (both from Thermo Fisher Scientific).

### Oxygen consumption rate and extracellular acidification rate

In all, 3 × 10^4^ cells/well were plated 16 h before the start of the experiment in growth media according to the cell line (as explained in Cell Culture and Reagents section). Oxygen consumption rate (OCR) and extracellular acidification rate (ECAR) were measured using an XFe96 extracellular flux analyzer (Seahorse Bioscience). Basal mitochondrial respiration was measured by the attainment of the initial OCR readings and subtraction of the OCR values after treatment with 10 μM antimycin A and 10 μM rotenone (Sigma-Aldrich). Maximal respiration was measured by the subtraction of the nonmitochondrial respiration by the maximum rate measurement after 1 μM carbonyl cyanide 4-(trifluoromethoxy) phenylhydrazone (FCCP) injection. Glycolysis was determined by subtraction of the last rate measurement before glucose injection by the maximum rate measurement before oligomycin injection. Glycolytic capacity was measured by subtraction of the last rate measurement before glucose injection by the maximum rate measurement after oligomycin injection. 2-Deoxyglucose (Sigma-Aldrich; 50 mM) was used to return ECAR to baseline. Experiments were performed in DMEM with no glucose or bicarbonate containing 2 mM glutamine (Sigma-Aldrich).

### RNAseq and qPCR

RNA was extracted using RNAeasy kit (Bio-Rad) and used to perform RNA-seq or to perform qPCR as previously described [30]. Sequences of primers used are presented in Supplementary Table 1. Libraries preparation, sequencing, and bioinformatics analysis of RNAseq were performed by Novogen (Novogen, Inc, Sacramento CA). Briefly, RNA integrity was assessed with Agilent Bioanalyzer 2100 to select RNA samples with RIN > 7.3 to 9.3. Two-hundred fifty to 300 base pair insert cDNA libraries, non–strand-specific, were prepared with New England Biolabs (Ipswich, MA) Next Ultra RNA Library Prep and sequenced with Illumina (San Diego, CA) HiSeq PE150 Platform ∼6G/sample Q30 > 90%. The reads were mapped to the human reference genome sequence using STAR v2.5 and v2.6.1, with a total mapping rate >90%/sample. For gene expression level analysis and to calculate the fragments per kilobase of transcript per million mapped reads, HTSeq v0.6.1 was used. The differential expression analysis between 2 different groups was done with DESeq2 R package v2_1.6.3. The *p*-values were adjusted using the Benjamini–Hochberg approach for controlling the false discovery rate, adjusted *P* < 0.05. TFCat and Cosmic databases were used to annotate the differential expressed gene. The enrichment analysis was done with cluster Profiler R package. The high-throughput sequencing data from this study have been published in GEO with the accession number GSE188774.

### RNA-seq data download and normalization method

The sample manifest along with the biospecimen sample details sheet and clinical details sheet for all RNA-Seq samples for the TCGA_LIHC (Liver cancer) project were downloaded from The Cancer Genome Atlas (TCGA) Data Portal (https://portal.gdc.cancer.gov) on 18th March 2021. Counts table for all the sample were downloaded using gdc-client 1.6 (https://gdc.cancer.gov/access-data/gdc-data-transfer-tool). The data were normalized as log CPM (counts per million) using edgeR, including TMM normalization [31].

### Subcellular fractionation

Fractionation experiments were performed as described before with some modification [32]. Briefly, 1 × 10^7^ cells were homogenized in a hand-held tight-fitting Teflon pestle homogenizer with 40 strokes in STM buffer (250 mM sucrose, 50 mM Tris–HCl pH 7.4, 5 mM MgCl_2_, protease and phosphatase inhibitor cocktail) and kept on ice for 30 min. The homogenate was centrifuged at 800 × *g* for 15 min to separate nuclear fraction as a pellet. The nuclear fraction was washed twice in STM buffer and resuspended in NET buffer (20 mM HEPES pH 7.9, 1.5 mM MgCl_2_, 0.5 M NaCl, 0.2 mM EDTA, 20% glycerol, 1% Triton-X-100, protease and phosphatase inhibitors). The supernatant was centrifuged at 11,000 × *g* for 10 min to separate the cytosolic fraction as supernatant and the mitochondrial faction as pellet. The mitochondrial pellet was washed once and resuspended in SOL buffer (50 mM Tris–HCl pH 6.8, 1 mM EDTA, 0.5% Triton-X-100, protease and phosphatase inhibitors). All steps were performed on ice and fractions were stored at −80 °C.

### Survival analysis of cancer patients with differentially expressed HKDC1

Kaplan–Meier Plotter (KM plotter, http://kmplot.com/analysis/) compiles publicly available data from repositories such as Gene Expression Omnibus (GEO), European Genome-Phenome Archive (EGA), and The Cancer Genome Atlas’ (TCGA). To examine the prognostic value of HKDC1 mRNA expression in LC, Pan-cancer RNA-seq database was used to evaluate the overall survival of cancer patients (*n* = 364). The two patient cohorts showing differential gene expression were compared by a Kaplan–Meier survival plot, and the hazard ratio with 95% confidence intervals and logrank *p*-values were calculated.

### TCGA dataset mining

Data from the publicly available TCGA dataset was mined using the websites (https://cistrome.shinyapps.io/timer/) and http://gepia2.cancer-pku.cn/#index.

### Transfection and stable cell-line generation

Transient transfection with HK2 and HKDC1 siRNA (Sigma; SASI_Hs02_0035783, SASI_Hs01_0008010) was employed to knockdown (KD) HK2 and HKDC1 protein expression in HepG2 and Hep3B2 cells. Experiments were conducted by plating cells in a six-well plate for next day transfection when cells where 70–80% confluent using Lipofectamine RNAiMax (Invitrogen; 13778100). A final concentration of (20 pmoL) siRNA was used in culture media without antibiotics overnight after which media was changed to normal growth media and cells were allowed to grow for another 24 h.

Lentiviruses to overexpress HKDC1-FL (pLV[Exp]-Bsd-EF1A > hHKDC1[NM_025130.4]/HA (VB210314-1076pxg) >10^8^ TU/mL) and HKDC1-TR (pLV[Exp]-Puro-CMV > (Human-HKDC1-TR)/HA (VB200213-1112yyx) >10^8^ TU/mL) were purchased commercially (VectorBuilder). Cells were infected with the 100 μL of the virus (in the presence of polybrene; 5 μg/mL) and selected with Blasticidin (10 μg/mL) for 15 days.

For doxycycline inducible shRNA-mediated HKDC1 knockdown, Hep3B2 cells were transfected with 4 μg of HKDC1 shRNA construct (Dharmacon; RHS4740-EG80201) using Lipofectamine LTX with Plus Reagent (Invitrogen; A12621). Forty-eight hours post transfection transfected cell were selected with puromycin (0.5 μg/mL) for 3 days. To examine the cells for RFP expression 24–48 h post transfection, 1 μg/mL doxycycline was used.

### Transmission electron microscopy

Cell pellets were fixed in a buffered solution of 2% paraformaldehyde + 2.5 % glutaraldehyde (pH 7.4), washed in 0.1 M Sorensen’s sodium phosphate buffer (SPB, pH 7.2) and post-fixed in buffered 1% osmium tetroxide for 1 h. After several buffer washes, samples were dehydrated in an ascending concentration of ethanol leading to 100% absolute ethanol, followed by two changes in propylene oxide (PO) transition fluid. Specimens were infiltrated overnight in a 1:1 mixture of PO and LX-112 epoxy resin, and 2 h in 100% pure LX-112 resin, and then placed in a 60 °C oven to polymerize (3 days). Ultra-thin sections (~75 nm) were cut (using a Leica Ultracut UCT model ultramicrotome), collected onto 200-mesh copper grids and contrasted with uranyl acetate and Reynolds’ lead citrate stains, respectively. Specimen were examined via JEOL JEM-1400F transmission electron microscope, operating at 80 kV. Digital micrographs were acquired using an AMT Biosprint 12M-B CCD Camera and AMT software (Version 7.01).

### Statistical analysis

For all in vitro experiments 3–8 replicates were done. For in vivo studies, a *n* = 8–10/group was chosen based on published studies and the animals were randomly assigned to different groups. Histological analysis from in vivo samples were analyzed blindly by the technician. Values are represented as means ± standard errors of the mean (SD). Data were analyzed either by student’s *t*-test or one-way/two-way ANOVA followed by a Tukey or Bonferroni post hoc test when applicable with equal variance between groups. Analysis of RNAseq data and enrichment analysis of DEG was performed by Novogene, Inc. Differentially regulated metabolites and enrichment analysis of metabolomics was performed with Metaboanalyst software. The statistical analyses were performed using GraphPad Prism 8 (GraphPad Software, La Jolla, CA). *p*-values less than 0.05 were considered significant.

## Results

### HKDC1 is overexpressed in LC

Supporting the observation that HKDC1 is overexpressed in LC [26, 33], we mined TCGA data, which showed that HKDC1 is significantly upregulated in human cancers (Supplementary Fig. 1A). We also show that HKDC1 has high expression in tumors of LC patients (Fig. 1A) with increased expression in different stages (I to III) of LC (Fig. 1B) and high HKDC1 expression is associated with lower survival in these patients (Fig. 1C). Further exploration also shows that HKDC1 is the hexokinase isoform most significantly upregulated in LC (Supplementary Fig. 1B). Further, since non-alcoholic fatty liver disease (NAFLD) is an independent risk factor for LC [3, 4], we used a mouse model to explore HKDC1 expression in a NASH-induced LC model (a NASH diet of high fat, cholesterol and sucrose diet (HF-HC-HSD) for 34 weeks). In the NASH diet group, 100% of mice developed NASH and 15% of that group developed LC in addition to NASH (NASH + LC). HKDC1 expression (mRNA and protein), was significantly higher (>10-fold) in the livers of the NASH diet fed mice as compared to controls (Fig. 1D; left panel). Moreover, mice that developed LC with NASH had >100-fold higher HKDC1 mRNA expression in the liver tissue (non-tumor regions) than controls (Fig. 1D; left panel), with corresponding enhanced HKDC1 protein expression (Fig. 1D; right panel). This progressive increase in hepatic HKDC1 expression from NASH to LC suggests an association of HKDC1 in the progression of liver disease to LC.

### HKDC1 expression is essential for LC growth and proliferation

**Fig. 2:**
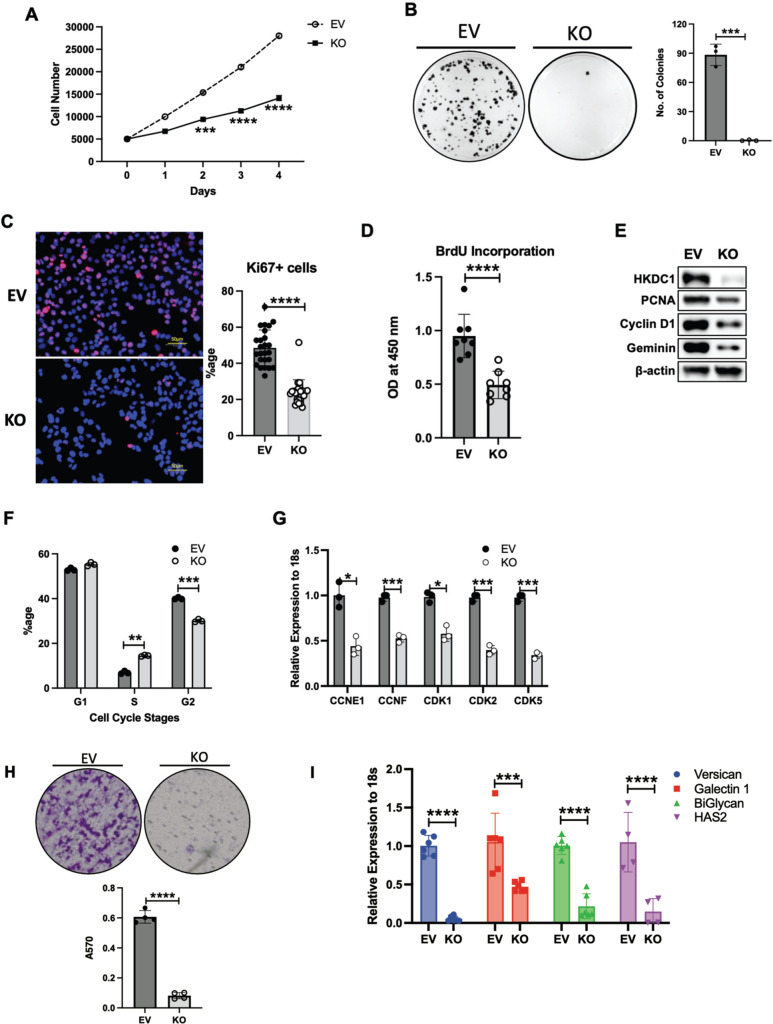

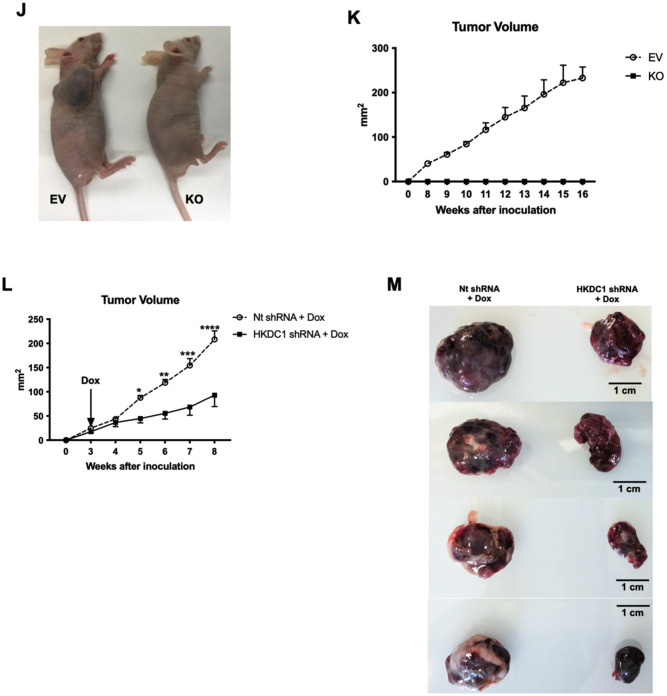
HKDC1 is essential for LC progression and survival. EV and HKDC1-KO cells (KO) were used for **A** cell proliferation assay, **B** colony-forming assay (left panel images representative of three images per group and right panel number of colonies were counted using Image J), **C** immunostaining for Ki67 proliferative marker (pink), nuclei are stained with DAPI (blue), **D** Cell proliferation was measured using the BrdU assay, **E** Western blot analysis was done in EV and KO cells for markers of cell proliferation and cell cycle (representative of two independent blots). **F** Cell-cycle analysis with propidium iodide, **G** mRNA expression by qPCR for cyclins and cyclin-dependent kinases. **H** In invasion assay, EV and KO cell suspensions (2 × 10^5^ cells/well) were added to the upper chambers and allowed to invade for 72 h. Invasive cells were stained with 0.1% crystal violet (upper panel) and were measured by enzyme-linked immunosorbent assay reader using 570 nm as test wavelength (lower panel). **I** mRNA expression by qPCR for proteoglycan synthesis genes. **J** In vivo tumor growth was assessed where 1 × 10^6^ EV or KO cells were inoculated into 4–6-week-old male Nu/J mice (*n* = 5), images were taken at endpoint (16 weeks post inoculation). **K** Tumor size was measured weekly till 16 weeks after appearance of tumor with a vernier caliper tumor weight till the end of the study. **L** Hep3B2 cells were transfected with shHKDC1 or (non-target) ntshRNA and transfected cells were selected with appropriate antibiotics, 1 × 10^6^ cells were inoculated into mice (*n* = 4). When tumors were visible, mice were given doxycycline (in diet) for 7 days to activate shRNAs. Tumor growth was measured weekly till 8 weeks after appearance of tumor with a vernier caliper. **M** Images of tumors at endpoint. All cell-line experiments (**A**–**G**) were performed 2–3 times with 3–5 replicates per experiment. Values are mean ± SD; *p < 0.05; **p < 0.01; ***p < 0.001; ****p < 0.0001 by Student’s *t*-test, or two-way ANOVA (for **K**).

Since HKDC1 has negligible expression in the adult liver [33, 34], and its expression is upregulated in LC [26, 27], we hypothesized that enhanced HKDC1 expression promotes LC proliferation. To test this hypothesis, we stably overexpressed HKDC1 in AML12 cells, a non-cancerous hepatocyte cell line, with minimal expression of HKDC1 (Supplementary Fig. 1C) and found that HKDC1 overexpression resulted in enhanced proliferation (Supplementary Fig. 1D) and survival (Supplementary Fig. 1E). Next, we selected 5 different LC cell lines with variable HKDC1 expression (Supplementary Fig. 2A) and downregulated HKDC1 expression using RNAi (Supplementary Fig. 2B), which inhibited cell survival (Supplementary Fig. 2C) in each cell line indicating that it is required for survival and proliferation. To further explore this, we developed a HKDC1 knockout (KO) cell line using Crispr-Cas9 in HepG2 cells (referred as HKDC1-KO from hereon) (Supplementary Fig. 2D, E). In these cells, HKDC1 ablation resulted in diminished proliferation and survival (Fig. 2A, B) as shown by reduced expression of proliferation marker, Ki67 (Fig. 2C), reduced bromodeoxyuridine (BrdU) incorporation (Fig. 2D) and proliferating cell nuclear antigen (PCNA; Fig. 2E).

Next, we analyzed gene expression in HKDC1-KO and control cells (carrying the empty vector; EV) by RNA-seq. HKDC1 ablation resulted in genome wide changes with >3000 genes significantly altered in HKDC1-KO cells (Supplementary Fig. 2F, G). Using gene ontology (GO) analysis, we found that 29 GO terms related to cell division machinery were downregulated (Supplementary Fig. 2H) consistent with the reduced proliferation rates in HKDC1-KO cells. Next, by cell-cycle analysis, HKDC1-KO cells have a significant increase of cells in synthesis (S) phase and decrease in G2 phase (Fig. 2F), which may be due to failure in DNA synthesis machinery or an intra-S-phase arrest to progress through this phase. Cyclins and cyclin-dependent kinases (CDKs) are major effectors of the cell cycle with key role in cancer [35, 36] where our data shows downregulation of key cell-cycle proteins such as cyclin D1 and Geminin (Fig. 2E) and mRNA levels of *CCNE1, CCNF, CDK1, CDK2* and *CDK5* (Fig. 2G).

Cell migration abilities are essential for cancer metastasis [37]. Therefore, we used the classical trans-well assay to assess the effect of HKDC1 ablation on cell invasive properties observing that HKDC1-KO cells lost the ability to migrate and invade (Fig. 2H). As cell migration depends on the synthesis of proteoglycans [38, 39], we searched for genes involved in proteoglycan synthesis in our RNA-seq data and found that they were significantly downregulated with HKDC1 ablation in HepG2 cells, which we then confirmed by qPCR (Fig. 2I).

**Fig. 3:**
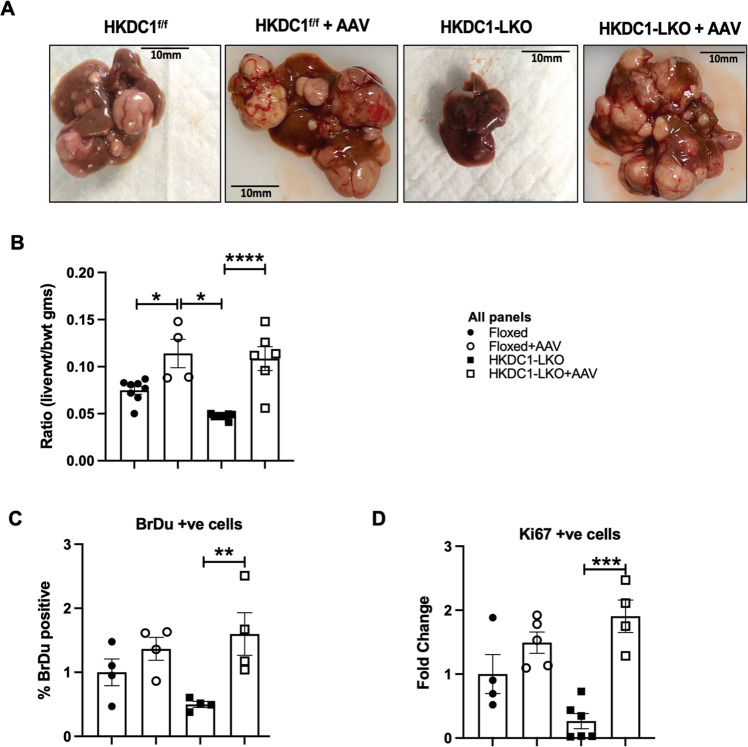
HKDC1 ablation impairs LC progression in an in vivo LC model. Two-week-old HKDC1^f/f^ and HKDC1-LKO male mice were injected with DEN (25 mg/kg). When mice were 8 weeks old, both groups were further divided into groups where one group received AAV expressing human-HKDC1 (HKDC1^f/f^ + AAV and HKDC1-LKO + AAV) and the AAV expressing null vector was used as the control with the two other groups (HKDC1^f/f^ and HKDC1-LKO), with  *n* = 3–7 per group. Ten months after DEN injection, mice were sacrificed. **A** Images of the livers are shown. **B** Livers were weighed and ratio to body weight was calculated. Liver sections were fixed, and immunohistochemistry was performed to show **C** BrdU and **D** Ki67-positive hepatocytes. Regions of tumor in the histology slides were identified and omitted from analysis. Values are mean ± SD; *p < 0.05; **p < 0.01; ***p < 0.001; ****p < 0.0001 by two-way ANOVA.

Next in an in vivo xenograft model (Nu/J mice) we injected EV and KO cells subcutaneously and observed that all (100%) mice injected with EV cells developed tumors and no mice injected with HKDC1-KO cells developed tumors (Fig. 2J–K and Supplementary Fig. 2I). Further we created an inducible shRNA-mediated knockdown of HKDC1 in Hep3B2 cells and performed xenografts experiments with these cell and control cells carrying scrambled shRNA (nt shRNA) (Supplementary Fig. 2J). Our data shows that although tumors form in both cell lines (containing scramble and shHKDC1), when the shRNA expression was induced to knockdown HKDC1 using a doxycycline diet, the tumors in shHKDC1 group of mice exhibited reduced growth as compared to cells carrying scrambled shRNA (Fig. 2L, M and Supplementary Fig. 2K). Next, in another model of LC, we used the classical chemical based (diethylnitrosamine; DEN) induction of LC with our liver-specific HKDC1 knockout mouse (HKDC1-LKO) model by crossing HKDC1 floxed (HKDC1^f/f^) mice [30, 33] with Albumin-Cre mice. DEN was injected at 14 days and the animals were observed for 40 weeks (Supplementary Fig. 3A–C). As a proof of concept, we re-expressed full-length human-HKDC1 (using AAVs) in a group of mice from both HKDC1^f/f^ and HKDC1-LKO groups (Supplementary Fig. 3A). At the terminal time point (40 weeks), we found that the HKDC1^f/f^ (controls) had a robust expression of HKDC1 (Supplementary Fig. 3B, C), larger livers and more tumors than HKDC1-LKO mice (Fig. 3A, B and Supplementary Fig. 3D). The mice from both control and HKDC1-LKO groups where HKDC1 was overexpressed had larger livers and more tumors (Fig. 3A, B and Supplementary Fig. 3D). Further, histological analysis revealed that livers from HKDC1-LKO mice with AAV treatment had the highest number of BrdU and Ki67-positive hepatocytes compared to all groups while HKDC1-LKO had the least number of positive cells compared to any other group (Fig. 3C, D). These data from both in vitro and in vivo LC models indicates that HKDC1 regulates several important cellular processes and establishes its role in the LC proliferation.

### HKDC1 ablation disrupts glucose metabolism

**Fig. 4:**
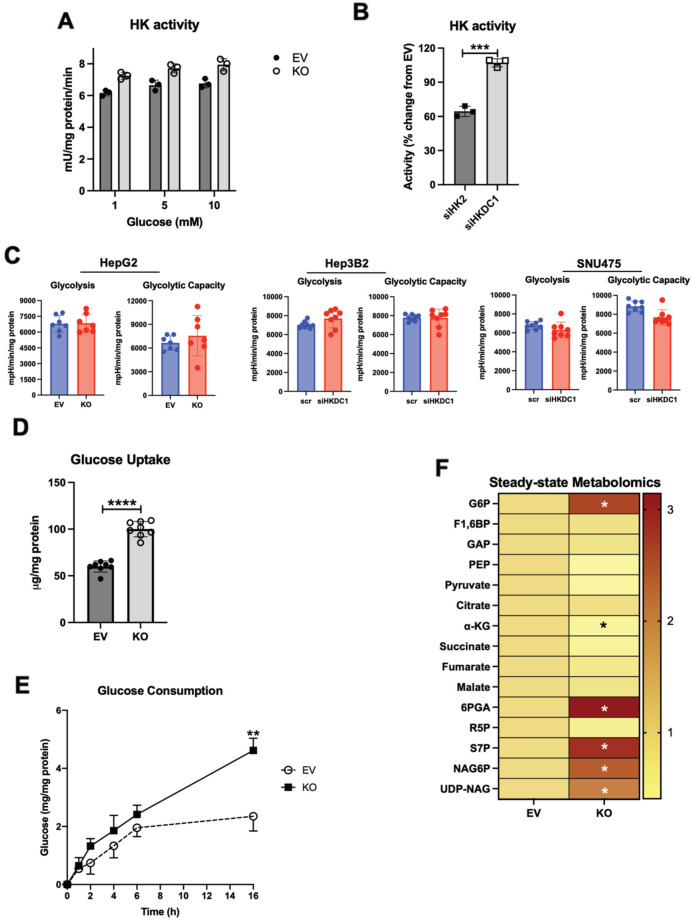

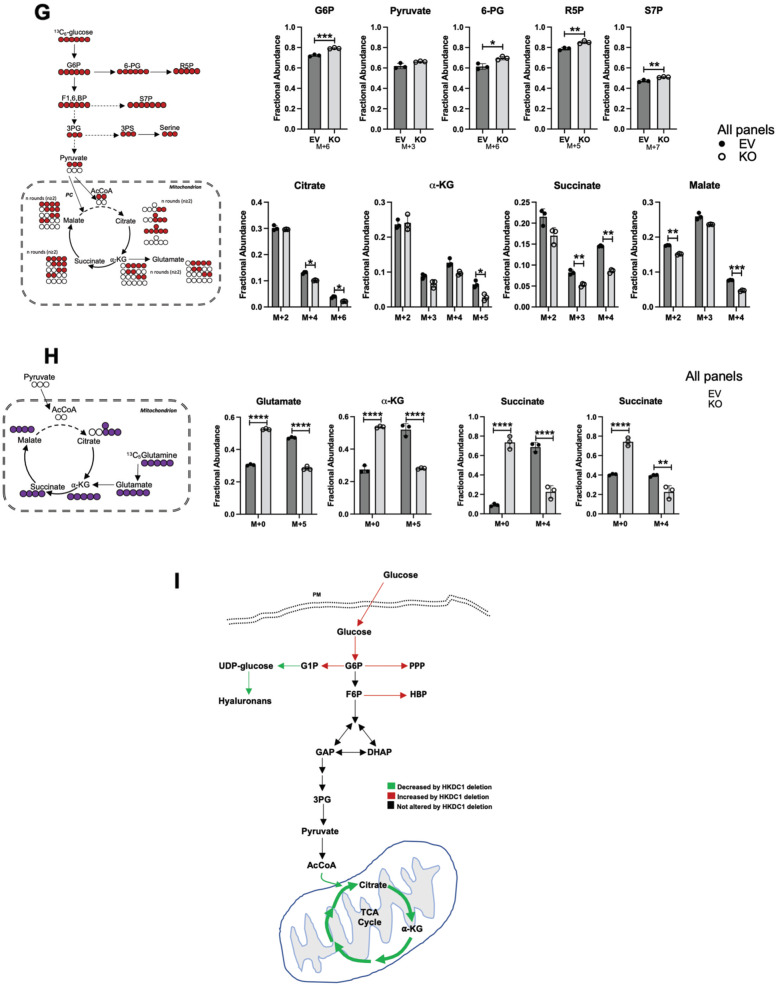
HKDC1-KO impairs glucose metabolism. **A** Hexokinase activity in EV and KO HepG2 cells. **B** HepG2 cells were treated with siRNA against either HK2 or HKDC1 for 24 h, cells were lysed, and hexokinase activity was assayed. **C** Seahorse metabolic analysis (ECAR) of EV and HKDC1-KO cells (left panel) and siRNA-mediated HKDC1 knockdown (siHKDC1) in Hep3B2 and SNU475 cells (center and right panels). **D** 2-NBDG (2-(N-(7-Nitrobenz-2-oxa-1,3-diazol-4-yl)Amino)-2-Deoxyglucose) fluorescent analog of glucose was used to assess glucose uptake in EV and KO HepG2 cells. **E** In EV and KO HepG2 cells, glucose consumption was assessed by measuring glucose concentration in media aliquots taken at designated time periods, which was subtracted from initial glucose concentration of media, obtaining glucose being consumed by the cells. **F** Steady-state metabolomics analysis of glycolytic and TCA cycle metabolites in cells cultured under standard growth condition (*n* = 3, independent biological replicates). **G** Mass isotopomer analysis of glycolytic and TCA cycle metabolites in cells cultured with 5.5 mM of [U-^13^C_6_] glucose and unlabeled glutamine (*n* = 3, independent biological replicates) for 4 h. **H** Mass isotopomer analysis of TCA cycle metabolites in cells cultured with 2 mM of [U-^13^C_5_] glutamine and unlabeled glucose (*n* = 3, independent biological replicates) for 4 h. **I** Schematic summarizing the changes in glucose flux upon HKDC1-KO. Exp **A**–**D** were performed 2–3 independent times, with three replicates per individual experiment. Values are ± SD; *p < 0.05; **p < 0.01; ***p < 0.001; ****p < 0.0001 by Student’s *t*-test (for **A**–**C**) or two-way ANOVA (for **D**–**F**).

Since HKDC1 is a putative HK gene, we next assessed the effect of HKDC1 ablation on other HKs, including their expression, cellular localization, and cellular HK activity. We show here that HKDC1 ablation had no effect on the protein or mRNA levels of the other HKs (Supplementary Fig. 4A, B). Further, total HK activity in the HKDC1-KO cells was not significantly different from EV cells (Fig. 4A). Since HK2 is also expressed in LC [12], we used siRNAs against either HK2 or HKDC1 to determine the relative impacts of these HKs on HK activity in HepG2 cells. Our data shows that when HK2 was knocked down (KD), there was ~40% reduction in cellular HK activity (Fig. 4B and Supplementary Fig. 4C) compared to no change when HKDC1 was KD (Fig. 4B and Supplementary Fig. 4C). Then, we measured extracellular acidification rate (ECAR) and our data shows that there is no change in glycolysis and glycolytic capacity in HepG2 and SNU-475 cells with HKDC1 knockdown while there was an increase in glycolysis in Hep3B2 cells. We also used AML12 cells with and without HKDC1 overexpression (OE) observing that HKDC1-OE had no effect on glycolysis or glycolytic capacity (Supplementary Fig. 4D). Overall, these data are consistent with our earlier studies, which show that HKDC1 is a poorly functioning HK (with low Km), as compared HK2 [33] and HK2 has a direct role in enhancing glycolysis [12].

Exploring other aspects of glucose metabolism, we observed that HKDC1 ablation significantly increases glucose uptake and consumption (Fig. 4D, E). We also observed that GLUT1 levels remain unchanged while surprisingly protein and mRNA levels of GLUT2, which is the predominant form in liver cells and a prognostic marker for LC [40] was reduced with HKDC1-KO (Supplementary Fig. 4E, F). Intriguingly levels of GLUT4, which is activated by the pro-survival PI3K-Akt pathway [41], was significantly upregulated (Supplementary Fig. 4E, F). Next, using steady-state metabolomics, focused on glucose metabolism, glucose-6-phosphate levels were increased in HKDC1-KO cells (Fig. 4F) without a similar increase in other glycolytic or TCA cycle metabolites; however, an increase in the levels of metabolites involved in the PPP and HBP shunts of the glycolysis occurred (Fig. 4F).

Going further using targeted metabolomics (U-^13^C_6_ labeled glucose), the HKDC1-KO cells, showed that labeled carbons from glucose are converted to glucose-6-phosphate (Fig. 4G; upper panel) but did not accumulate in the glycolytic intermediates, and rather cause an increase in PPP and HBP metabolites (Fig. 4G; upper panel). Moreover, there was a significant decrease in labeled glucose carbons entering the TCA cycle (Fig. 4G; lower panel). Since anaplerosis feeds metabolites to the TCA cycle [42] in addition to glucose, we performed a U-^13^C_5_ labeled glutamine targeted metabolomics observing that HKDC1 ablation has a significant decrease in labeled TCA cycle metabolites upon HKDC1-KO (Fig. 4H; all panels). In a rescue experiment, we assessed whether addition of glucose and TCA metabolites could rescue HKDC1-KO cells by supplementation the growth media with cell permeable forms of pyruvate (methyl pyruvate) and alpha-ketoglutarate (octyl-alpha-ketoglutarate), however, metabolite supplementation did not rescue cell proliferation upon HKDC1 ablation (Supplementary Fig. 4G). As summarized in Fig. 4I, cells lacking HKDC1 have enhanced glucose consumption fueling the PPP and HBP pathways but decreased TCA cycle flux.

### HKDC1 is essential for mitochondrial function

**Fig. 5:**
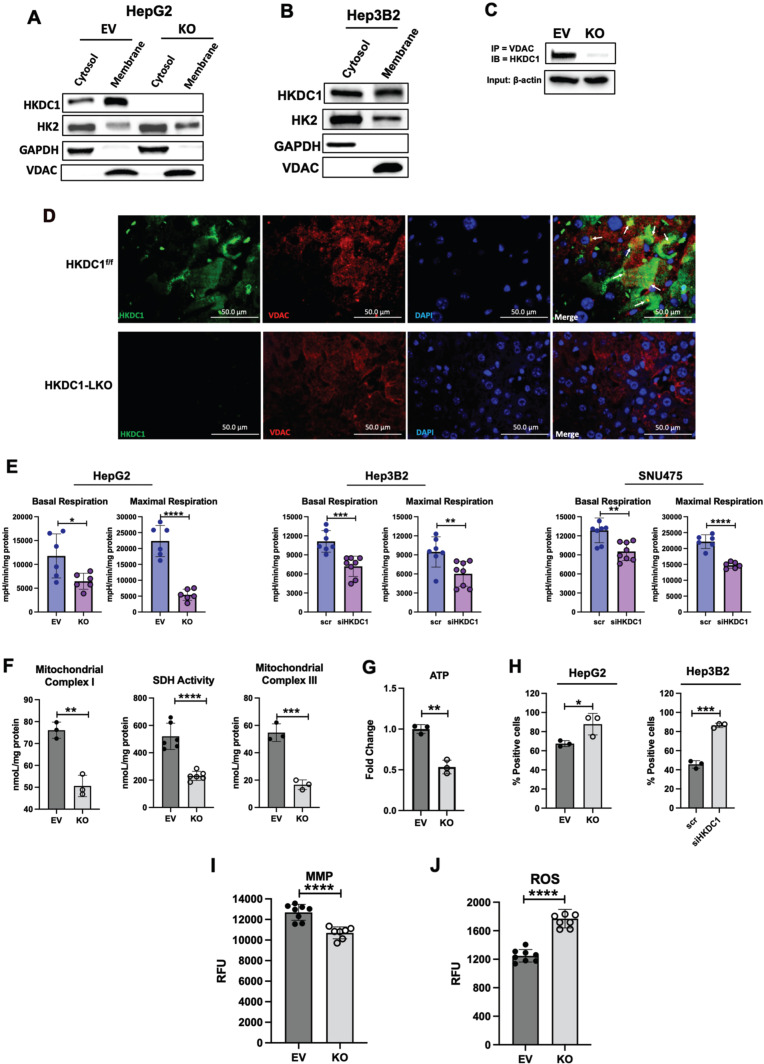

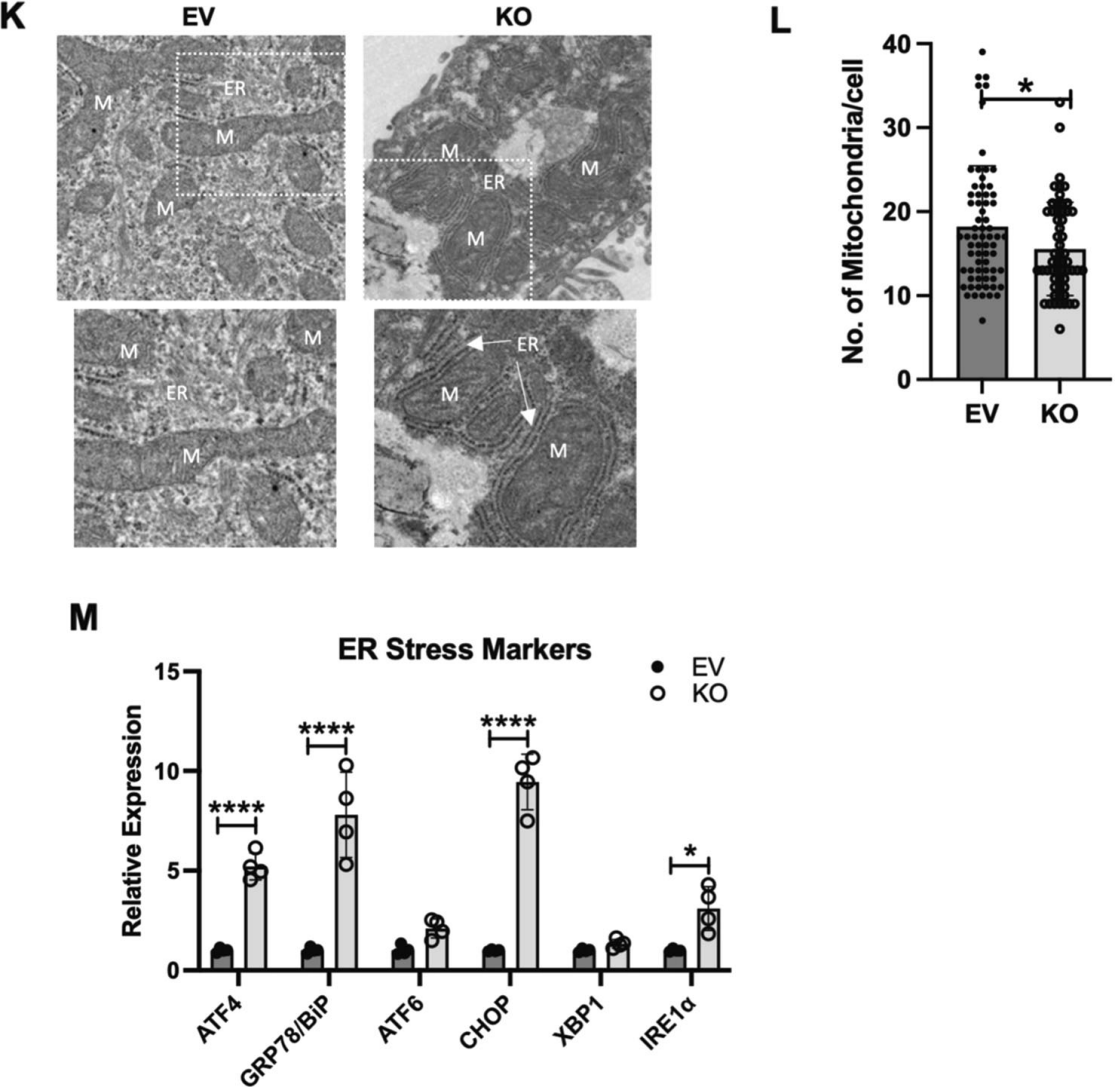
HKDC1 is essential for mitochondrial function in LC. Cell fractionation experiment showing cytosolic, membrane and nuclear fractions in **A** EV and KO HepG2 cells and **B** Hep3B2 cells. **C** EV and KO HepG2 cells were used in co-immunoprecipitation experiments where IP was done with anti-VDAC antibody and then immunoblotting (IB) was done with HKDC1 antibody. **D** Liver sections from DEN treated HKDC1^f/f^ and HKDC1-LKO mice were used to show HKDC1-VDAC interaction by immunohistochemical staining of HKDC1 and VDAC (images are representative of *n* = 3 per group). **E** Seahorse metabolic analysis (OCR) of EV and HKDC1-KO cells and siRNA-mediated HKDC1 knockdown (siHKDC1) in Hep3B2 and SNU475 cells (center and right panels). **F** Activity assay for mitochondrial complex I (left panel), SDH (center panel) and complex III (right panel) of EV and HKDC1-KO cells. **G** Intracellular ATP levels relative to EV (fold-change) (*n* = 3). **H** Mitochondrial Ca^2+^ levels were assessed by Rhod-2AM fluorescence using flow cytometry of EV/HKDC1-KO cells (left panel) and Hep3B2 cells treated with siRNA against HKDC1 for 24 h (right panel). 50,000 cells were assessed for each sample, and data was plotted on bar graphs with statistics (*n* = 3) for each cell line. **I** Mitochondrial membrane potential was measured by TMRE fluorescence. **J** Intracellular ROS levels are shown. **K** HepG2 expressing empty vector (EV) or HKDC1 knockout (KO) cells were processed for TEM. In all, 20–25 images were taken for each cell at different magnifications. Images shown here were taken at 4000x. Inset (shown in white) was enlarged (below) to show mitochondria and ER (M = mitochondria, ER = endoplasmic reticulum). **L** Mitochondria were counted for each cell (20–27 cells per sample) by Image J software. All cell-line experiments were performed 2–3 independent times with 3–8 replicates per experiments. **M** qPCR analysis in EV and KO cells to assess mRNA levels of ER stress markers. Values are ±SD; *p < 0.05; **p < 0.01; ***p < 0.001; ****p < 0.0001 by Student’s *t*-test (for **B**, **E**–**G**, **I**) or 2 way ANOVA (for **C**, **J**).

We have previously shown that HKDC1 localizes to the mitochondria and binds with mitochondrial proteins like VDAC [33], to further confirm this we performed subcellular fractionation with modulated HepG2 cells, and observed that HKDC1 was predominantly in the membrane fraction with a lesser amount in the cytosol (Fig. 5A). In contrast, HK2, which has been shown to bind to the mitochondria in some cancer cell lines [17, 18], was found to be in the cytosol and membrane fractions (Fig. 5A). Interestingly, in HKDC1-KO cells, we did not see more HK2 in the mitochondrial fraction, suggesting that HKDC1 may not compete with HK2 for mitochondrial binding at these cells (Fig. 5A). We confirmed this observation by examining two other LC cell lines (Fig. 5B and Supplementary Fig. 5A). Furthermore, we performed co-immunoprecipitation studies showing that HKDC1 and VDAC colocalize in EV cells, whereas this interaction is absent in HKDC1-KO cells (Fig. 5C). Lastingly, in liver sections from DEN treated HKDC1^f/f^ and HKDC1-LKO mice by immunohistochemistry, we observed that HKDC1 and VDAC partially colocalize in HKDC1^f/f^ liver (Fig. 5D) but not in HKDC1-LKO liver.

Mitochondrial function is essential for carcinogenesis [15, 16, 43] and our RNA-seq data shows that several downregulated GO terms (cellular component) upon HKDC1-KO belong to the mitochondria (Supplementary Fig. 5B); thus, HKDC1 might be required for optimal mitochondrial function in LC. To investigate this, we performed Seahorse analysis three LC cells lines observing that both basal and maximal respiration was significantly reduced in HKDC1-KO/KD cells. These data suggest that HKDC1 is important for mitochondrial function (Fig. 5E). Next, we measured activities of mitochondrial complex I, SDH and III observing that HKDC1-KO significantly reduces the activities of all three complexes (Fig. 5F). Energy in the form of ATP mainly comes from mitochondrial activity and enhanced glucose consumption observed in HKDC1-KO cells is not sufficient to maintain ATP levels resulting in significantly reduced ATP levels and AMPK activation in HKDC1-KO cells (Fig. 5G and Supplementary Fig. 5C, D).

Calcium (Ca^2+^) influx from the ER is essential for mitochondrial metabolism’ therefore, we assessed mitochondrial Ca^2+^ and found that HKDC1-KO/KD cells had significantly higher levels of Ca^2+^ (Fig. 5H). High mitochondrial Ca^2+^ also leads to decreased membrane potential (MMP) and induces reactive oxygen species (ROS) production, we assessed both and found that HKDC1-KO cells had decreased MMP (Fig. 5I) and enhanced ROS production (Fig. 5J) [44–47]. Investigating further, we used transmission electron microscopy to examine ER-mitochondrial appositions and structure in HKDC1-KO cells. We observed that HKDC1-KO cells displayed a markedly higher degree of ER-mitochondria contact sites and lower hepatic mitochondrial number (Fig. 5K). We also observed that mitochondria from HKDC1-KO cells were rounder and swollen compared to the tubular mitochondria observed in EV cells (Fig. 5K); however, a three-dimensional reconstruction is needed to confirm these observations. These data suggests that higher mitochondrial Ca^2+^ is associated with mitochondrial dysfunction in HKDC1-KO cells.

Increased mitochondria-ER contact could be due to mitochondrial dysfunction, ER stress or both; therefore, we again analyzed our RNA-seq data for GO terms related to ER stress and observed that 28 out of 30 significantly upregulated GO terms (biological process) in HKDC1-KO cells (Supplementary Fig. 5F). We further confirmed these findings using qPCR for ER stress markers including ER chaperone protein BiP (binding-immunoglobulin protein aka GRP-78), activating transcription factor 4 (ATF4) and C/EBP-homologous protein (CHOP), which are the effectors of PKR-like ER kinase (PERK) (Fig. 5M) [48], and inositol requiring enzyme 1α/β (IRE1) [48] (Fig. 5M). Therefore, we show here that when HKDC1 is not bound to the mitochondria (HKDC1-KO), there is increased mitochondria-ER interaction, ER stress and mitochondrial dysfunction, which may result in altered metabolism and cell death.

### Mitochondrial interaction of HKDC1 is essential for its role in LC progression

**Fig. 6:**
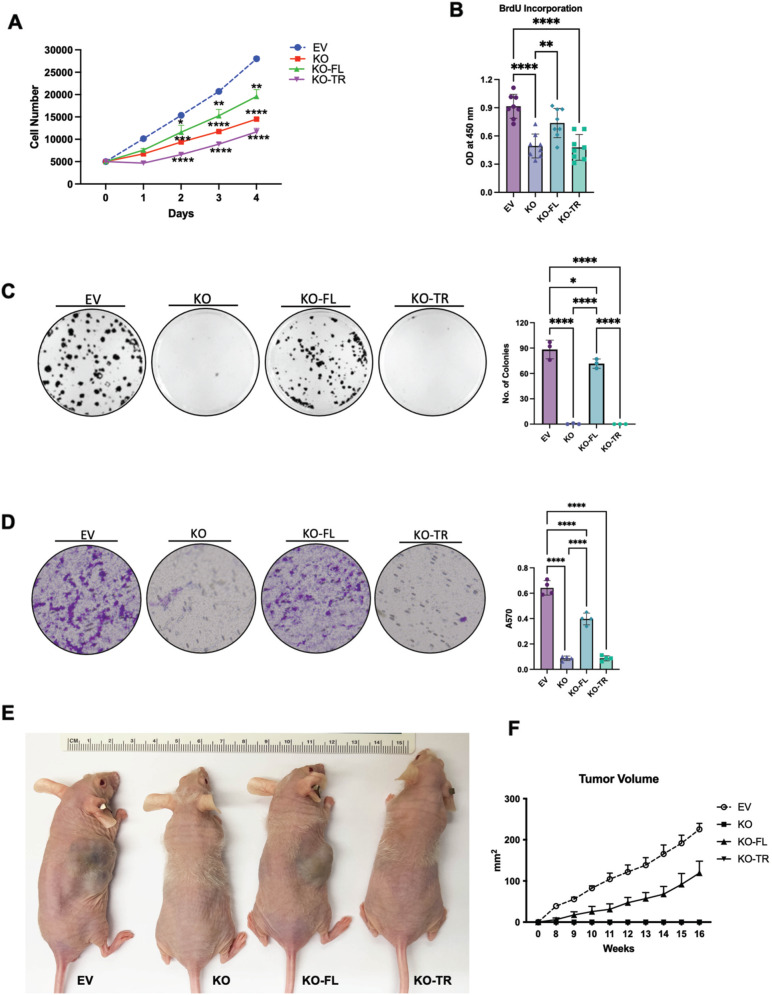

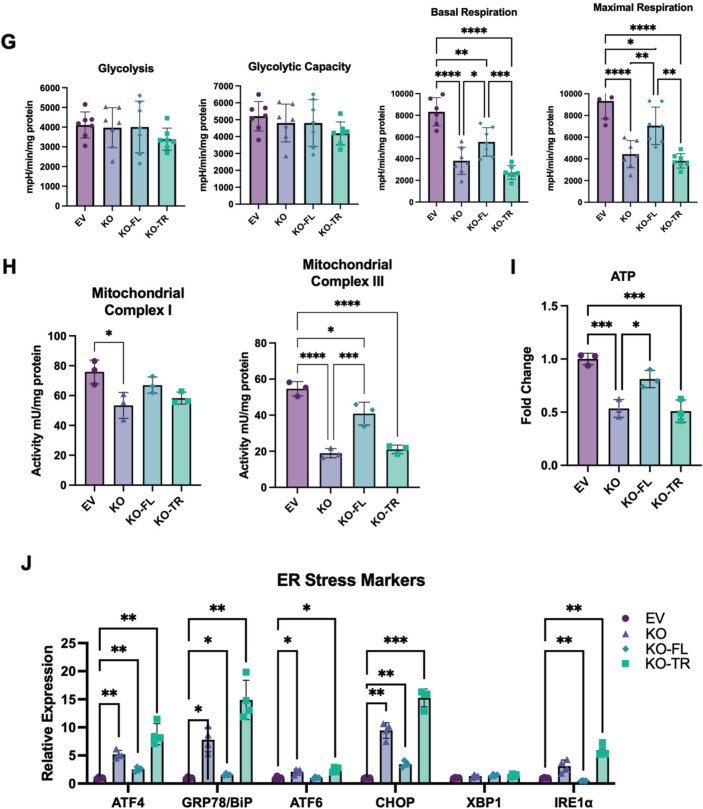
HKDC1-mitochondria interactions are necessary for mitochondrial function in LC. Using the EV, HKDC1-KO, KO-FL, and KO-TR cells, the following experiments were performed; **A** cell proliferation assay, **B** cell proliferation was measured using the BrdU assay, **C** colony formation assay (left panel images representative of three images per group and right panel number of colonies were counted using Image J software), **D** in invasion assay 2 × 10^5^ cells/well were added to the upper chambers and allowed to invade for 72 h. Invasive cells were stained with 0.1% crystal violet (representative images; left) and were measured by enzyme-linked immunosorbent assay reader using 570 nm as test wavelength (quantification; right panel), **E** in vivo tumor growth was assessed where 1 × 10^6^ EV or KO or KO-FL or KO-TR cells were inoculated into 4–6-week-old male Nu/J mice (*n* = 4–6), images were taken at endpoint (16 weeks post inoculation), **F** tumor size was measured weekly till 16 weeks after appearance of tumor with a vernier caliper tumor weight till the end of the study, **G** seahorse metabolic analysis (ECAR and OCR), **H** activity assay for mitochondrial complex I (left panel) and complex II (right panel) for each cell lines was also performed, **I** Intracellular ATP levels relative to EV (fold-change) (*n* = 3) and **J** qPCR analysis to assess mRNA levels of ER stress markers. All cell-line experiments were performed 2–3 independent times with at least three replicates per experiments. Values are mean ± SD; *p < 0.05; **p < 0.01; ***p < 0.001; ****p < 0.0001 by one-way ANOVA (for **B**–**D**) and two-way ANOVA.

Since HKDC1 ablation inhibits LC proliferation and also induces mitochondrial dysfunction, we questioned whether mitochondrial interaction of HKDC1 is necessary for its role in LC progression. To investigate this, we used a truncated version of HKDC1 lacking the first 20 amino acids essential for mitochondrial binding (HKDC1-TR), compared to full-length HKDC1 (HKDC1-FL). To avoid the involvement of endogenous HKDC1, we used HKDC1-KO cells to overexpress HKDC1-TR and HKDC1-FL (Supplementary Fig. 6A, B). Moreover, we confirmed with fractionation experiment (Supplementary Fig. 6C) and co-IP (Supplementary Fig. 6D) that HKDC1-TR did not localize at the mitochondria. Using these cells lines in proliferation and survival assays, our data shows that HKDC1 ablation induced reduction in proliferation, survival, and invasion was significantly rescued by HKDC1-FL overexpression in both in vitro and in vivo (xenografts) assays but not by HKDC1-TR (Fig. 6A–F). In Seahorse assay, HKDC1-FL overexpression was able to enhance both basal and maximal respiration compared to HKDC1-KO whereas HKDC1-TR overexpression remained similar to KO cells (Fig. 6G). We observed a similar trend on mitochondrial complex activities where HKDC1-FL overexpression enhanced both complex I and III activities compared to KO cells and the TR version was similar to KO cells (Fig. 6H) and also a similar increase in ATP levels in KO-FL cells (Fig. 6I). Finally, we checked for ER stress markers that were upregulated in HKDC1-KO cells and our data shows that the expression of these markers was restored in HKDC1-FL cells but the expression either remained similar to HKDC1-KO cells or were further upregulated in HKDC1-TR cells (Fig. 6J). This confirms our hypothesis that mitochondrial interaction of HKDC1 is essential for its role in LC.

## Discussion

Here we show that HKDC1 deletion in human LC cells inhibits proliferation and tumorigenesis both in vitro and in vivo and hepatocarcinogenesis in mice. While HKDC1 ablation does not change cellular HK activity, it induces glucose uptake with enhanced flux through the glycolysis shunt pathways PPP and HBP, and reduced TCA cycle metabolites. Additionally, we show an increased expression of GLUT4, which seems contradictory as GLUT4 is under the control of the PI3K-Akt pathway. While this pathway is considered pro-tumorigenic, Akt also has pro-survival functions [49, 50] therefore, increased GLUT4 expression could be a pro-survival mechanism in HKDC1-KO cells.

Further mitochondrial binding of hexokinases has been suggested to be necessary for cancer progression [13, 51]. We provide evidence here for the first time that HKDC1 interaction with the mitochondria is essential, and its deletion induces mitochondrial dysfunction in LC. We further prove this by using a mitochondrial binding deficient HKDC1 (HKDC1-TR), which when re-expressed in HKDC1-KO cells cannot restore mitochondrial function. As cells make most of their energy in the form of ATP from mitochondrial function, HKDC1-KO cells had significantly less ATP than control cells. This decrease in cellular ATP might result in a metabolic stress where cells then resort to taking in more glucose to meet the energetic demands of the cell. However, since HKDC1-KO cells have impaired mitochondrial function, enhanced glucose flux resulting from increased uptake does not fuel the TCA cycle but is moved to glucose shunt pathways. As cancer cells in the synthetic (S) phase of the cell cycle need ATP to prepare the cells for division, a lack of ATP might result in cell-cycle arrest as observed in HKDC1-KO cells, however, this needs further mechanistic investigation.

Mitochondrial dysfunction induces ER stress due to reduced ATP levels in the cell [52] and we found that HKDC1 ablation also leads to more ER stress. ER stress is a primary mechanism for cell survival, however, if it is persistent and unmitigated, the cell can switch the signaling in favor of cell death [48, 53–55] or it could induce cell-cycle arrest in the synthetic phase [56–58]. Our data shows that HKDC1-KO cells are arrested in the S-phase, and we hypothesize that HKDC1-KO-mediated metabolic/energetic stress elevates ER stress, which could be a probable mechanism explaining this cell-cycle blockade. We also show that upon HKDC1-KO, there is increased ER-mitochondria contact sites, leading to mitochondrial Ca^2+^ overload and abnormal mitochondrial structure. Ca^2+^ regulates mitochondrial function in a complex manner, which is incompletely understood where transient increase in mitochondrial Ca^2+^ can stimulate mitochondrial function [59, 60] and sustained ER-mitochondrial Ca^2+^ accumulation leads to mitochondrial dysfunction and increased ROS generation [61]. Our data clearly shows that HKDC1-KO leads to mitochondrial dysfunction; however, our study does not specifically address whether ER stress is a consequence of mitochondrial function or vice versa. Future studies are warranted to mechanistically investigate this phenomenon.

To summarize, mitochondrial bound HKDC1 regulates metabolism, proliferation, and survival of LC and HKDC1-KO significantly affects glucose flux, energy metabolism and mitochondrial function leading to less ATP thereby impacting cell-cycle progression and ER stress induction. Since HKDC1 has nominal expression in normal hepatocytes, but is highly upregulated in LC cells, novel small molecule and peptide-based inhibitors could be designed to target HKDC1, specifically its mitochondrial interaction in LC. Although our current study brings forward a previously unknown player (HKDC1) in LC progression, it also raises many questions that need to be addressed in future studies. Some of these questions are how is HKDC1 involved in regulation of cyclins/CDKs and thereby cell-cycle regulation. Also, since CDKs are also shown to play important roles in energy metabolism, it may also be possible that HKDC1 ablation mediated metabolic reprogramming in LC may be due to changes in CDK expression in addition to mitochondrial dysfunction. Our results also show that upon HKDC1 ablation there is enhanced glucose uptake due to increased GLUT4 expression. Since GLUT4 is controlled by the PI3K-Akt pathway, which has both tumorigenic and pro-survival roles, it will be important to investigate how HKDC1 ablation affects pro-survival strategies in LC. Finally, our work clearly shows that HKDC1 is involved in progression of LC, but it remains to be investigated whether it is also essential for cancer metastasis.

## Supplementary information

Supplementary Info

Supplementary Figures

Previous Rev Version

Original Data File

Reproducibility checklist

New Co-author addition agreement

Author Contribution Form

## Data Availability

The authors confirm that the data supporting the findings of this study are available within the article [and/or] its supplementary materials. TCGA data was derived from the following resources available in the public domain: [http://www.cbioportal.org]. RNA-seq data is available at GSE188774.
