# Supplementary material for: The hexokinase “HKDC1” interaction with the mitochondria is essential for liver cancer progression
Source: Cell Death Dis. 2022 Jul 28;13(7):660. doi: 10.1038/s41419-022-04999-z (PMC9334634; doi:10.1038/s41419-022-04999-z)
Supplement: Supplementary file 1 — Supplementary Info [file 41419_2022_4999_MOESM1_ESM.docx]

**Supplementary Fig Legends**

**Supplementary Fig 1.** **A)** mRNA expression data of HKDC1 in various types of cancer in human patients were mined from the The Cancer Genome Atlas (TCGA) data set using the Timer website (<https://cistrome.shinyapps.io/timer/>). Supplementary Table 3 list all abbreviations for the tumor types included. **B)** Differential expression of HK1-2, GCK and HKDC1 in LIHC patients from TCGA data set (as described in methods) was mined; Tumor (n=369) and normal surrounding tissue (n=50). **C)** HKDC1 was overexpressed using lentivirus (LV) in AML12 cells; top panel (immunoblot) and bottom panel (qPCR) showing overexpressed HKDC1 protein and mRNA levels. **D)** Cell proliferation curves in modulated AML12 cells, EV: cells with empty vector; OE: cells overexpressing HKDC1. **E)** Colony forming assay, EV: cells with empty vector; HKDC1: cells overexpressing HKDC1. All cell line experiments (**C-E**) were performed 2-3 times with 3-8 replicates per experiment. Values are ± SEM; **p < 0.01; ***p < 0.001; ****p < 0.0001 by Wilcoxon signed-rank test (for 1A), 2-way ANOVA (for 1B) and students t-test (for 1C-D).

**Supplementary Fig 2.** **A)** HKDC1 mRNA expression in a panel of human LC cell lines as compared to expression of 18s in each cell line. **B)** siRNA mediated HKDC1 knockdown in LC cell lines. Cells were transfected with siRNAs against *HKDC1* (siHKDC1) or scramble (scr) for 48h following which cells were harvested and subjected to immunoblot (left panel for HepG2 cells) and qPCR (right panel for HepG2, Hep3B2, Huh7, SNU-485 and SNU-387) to assess HKDC1 protein and mRNA levels. **C)** Colony forming assay in the panel of LC cell lines after siRNA mediated knockdown of HKDC1. **D)** Crispr-Cas9 was used to knockout HKDC1 in HepG2 cells; top panel shows protein expression of HKDC1 after sgRNA transfection and bottom panel shows mRNA expression. **E)** Genomic sequencing in the KO pool (see methods) was done to provide evidence for Crispr-cas9 mediated genomic alteration of *HKDC1* (left panel EV; right panel KO). **F)** Volcano plot created from list of differentially expressed genes from RNA-seq data. **G)** Unique and overlapping expressed genes in EV and KO cells (from RNA-seq data analysis). **H)** Gene ontology (GO) terms plotted for downregulated GO terms (from RNA-seq data analysis). **I)** *in vivo* tumor growth. 1X10^6^ EV or KO cells were inoculated into mice (n=4-5). Images of mice bearing tumors were taken at endpoint. **J)** Hep3B2 cells were transfected with shHKDC1 (activated by doxycycline treatment) or (non-target) ntshRNA and transfected cells were selected with appropriate antibiotics. Cells were then treated with doxycycline for 3 days and HKDC1 protein and mRNA expression was assessed by immunoblot (left panel) and qPCR (right panel). **K)** Hep3B2 cells were transfected with shHKDC1 or (non-target) ntshRNA and transfected cells were selected with appropriate antibiotics, 1X10^6^ cells were inoculated into mice (n=4). When tumors were visible, mice were given doxycycline (in diet) for 7 days to activate shRNAs. Tumor growth was measured weekly till 8 weeks after appearance of tumor with a vernier caliper. Images of mice bearing tumors were taken at endpoint. All cell line experiments (**A-D**) were performed 2-3 times with 3-8 replicates per experiment. Values are ± SEM; ***p < 0.001; ****p < 0.0001 students t=test (for 2D) and 2-way ANOVA (for 2B, J).

**Supplementary Fig 3.** **A)** Scheme for DEN treated mice. 14d male pups were injected with 25m/kg DEN intra-peritoneally. When mice were 8 weeks old, mice form both groups were further divided into groups where one group (n=3) received AAV-Null and the other AAV-HKDC1-FL. Mice were monitored till 40 weeks and sacrificed at that time point. **B)** mRNA expression of *HKDC1* from normal and tumor from liver tissue (n=3). **C)** Immunoblot to assess protein expression of HKDC1 in normal and tumor samples of HKDC1^f/f^ mice liver sample (n=2). **D)** Representative images from DEN induced tumorigenesis experiment. Two-week-old HKDC1^f/f^ and HKDC1-LKO male mice were injected with DEN (25 mg/kg). When mice were 8 weeks old, both groups were further divided into groups where one group received AAV expressing human HKDC1 (HKDC1^f/f^+AAV and HKDC1-LKO+AAV) and the AAV expressing null vector was used as the control with the two other groups (HKDC1^f/f^ and HKDC1-LKO), with N=4-7 per group. Values are ± SEM; ****p < 0.0001 by 2-way ANOVA.

**Supplementary Fig 4.** EV and KO HepG2 cells were analyzed for expression of different HKs by **A)** immunoblot and **B)** qPCR. **C)** HepG2 cells were treated with siRNA against either HK2 or HKDC1 for 24h, cells were lysed, qPCR analysis (relative expression to scramble siRNA (scr)) showing siRNA efficiency. **D)** Seahorse metabolic analysis (ECAR) of AML-12 cells expressing either empty vector (EV) or full-length HKDC1 (OE-HKDC1-FL). **E)** qPCR analysis in EV and KO cells showing the expression level of *GLUT1*, *GLUT2* and *GLUT4*. **F)** Immunoblot showing expression of GLUT2 and GLUT4 in EV and KO cells. **G)** EV and KO cells were incubated with 1mM methyl pyruvate (MP) or 200 uM octyl-alpha ketoglutarate (o-aKG) for 24 hours followed by MTT assay. All cell line experiments were performed 2-3 times with 3-5 replicates per experiment. Values are ± SD; **p < 0.01; ***p < 0.001 by student’s t-test (for 4C) or 2-way ANOVA (for 4B and E).

**Supplementary Fig 5.** **A)** Subcellular fractionation of Huh7 cells lines followed by immunoblot to show HKDC1 expression in cytosolic and membrane compartments. **B)** Gene ontology (GO) terms plotted for significantly downregulated GO terms in cellular component (from RNA-seq data analysis). **C)** Relative levels in fold change (to EV) of ATP, ADP and AMP values from steady-state metabolomics (n=3). **D)** immunoblot of EV and KO showing expression of AMPK (phosphorylated and total; immunoblot is representative of 2 different blots). **E)** immunoblot of EV and KO showing expression of mitochondrial proteins; right panels show densitometry (immunoblot is representative of 3 different blots). **F)** Gene ontology (GO) terms plotted for significantly upregulated GO terms in biological processes (from RNA-seq data analysis). Values are ± SEM; *p < 0.05; **p < 0.01 by student’s t-test.

**Supplementary Fig 6.** HKDC1-KO (KO) HepG2 cells were used to overexpress HA-tagged HKDC1-FL (KO-FL) or HKDC1-TR (KO-TR) by lentiviruses **A)** mRNA expression (n=3) and **B)** protein expression was analyzed after cells stably transduced cells were selected with appropriate antibiotics. **C)** Subcellular fractionation of KO-FL and KO-TR cells followed by immunoblot to show that only HKDC1-FL and not HKDC1-TR localizes at the membrane. **D)** KO-FL and KO-TR HepG2 cells were used in co-immunoprecipitation experiments where IP was done with anti-VDAC antibody and then immunoblotting (IB) was done with HA antibody. **E)** *in vivo* tumor growth. 1X10^6^ EV or KO or KO-FL or KO-TR cells were inoculated into mice (n=4-6). The * indicates a mouse that died one week before scheduled sacrifice time. Images of mice bearing tumors were taken at endpoint. All cell line experiments were performed 2-3 times with 3-5 replicates per experiment. All immunoblots are representative of 2-3 different blots. Values are ± SEM; **p < 0.01; ***p < 0.001, ****p < 0.0001 by one-way ANOVA (for 6A, D)

**Supplementary Table 1: List of Primers**

| Gene | Species | Forward Primer | Reverse Primer |
| --- | --- | --- | --- |
| *ATF4* | Human | TGCTACAGGGGAATGACT | TGCTACTGCAGGGGAATGACT |
| *ATF6* | Human | AATATATGCTAGGGTTAGAGGC | TTCTCTGACACAACTTCATC |
| *BIGLYCAN* | Human | GGTGGTCTATCTGCACTCCAA | GGCTGATGCCGTTGTAGTAGG |
| *CCNE1* | Human | AAGGAGCGGGACACCATGA | ACGGTCACGTTTGCCTTCC |
| *CCNF* | Human | CACAAAGCATCCATATTGCACTG | TGGTCAGACATCCCTGATGAG |
| *CDK1* | Human | GGATGTGCTTATGCAGGATTCC | CATGTACTGACCAGGAGGGATAG |
| *CDK2* | Human | GTACCTCCCCTGGATGAAGAT | CGAAATCCGCTTGTTAGGGTC |
| *CDK5* | Human | TTTTTCCCGGCAATGATGTCG | GCAGCTTGGTCATAGAGGGC |
| *CHOP* | Human | ACCAAGGGAGAACCAGGAAACG | TCACCATTCGGTCAATCAGAGC |
| *GALECTIN1* | Human | TCGCCAGCAACCTGAATCTC | GCACGAAGCTCTTAGCGTCA |
| *GCK* | Human | GCAGAAGGGAACAATGTCGTG | CGTAGTAGCAGGAGATCATCG |
| *GLUT1* | Human | CATCATCTTCATCCCGGC | CTCCTCGTTGCGGTTGAT |
| *GLUT2* | Human | TACATTGCGGACTTCTGTGG | AGACTTTCCTTTGGTTTCTGG |
| *GLUT4* | Human | TGGGCTTCTTCATCTTCACC | GTGCTGGGTTTCACCTCCT |
| *GRP78/BiP* | Human | CGGGCAAAGATGTCAGGAAAG | TTCTGGACGGGCTTCATAGTAGAC |
| *HAS2* | Human | TCCTGGATCTCATTCCTCAGC | TGCACTGAACACACCCAAAATA |
| *HK1* | Human | CACATGGAGTCCGAGGTTTAT | CGTGAATCCCACAGGTAACTTC |
| *HK2* | Human | GAGCCACCACTCACCCTACT | CCAGGCATTCGGCAATGTG |
| *HKDC1* | Mouse | CCACTGCCTCTGTGAAGATG | GACCCGAAACTTGGATCCTC |
| *HKDC1* | Human | GAATCCTGGCAAGCAGAGATACG | GATCAGGATCTGCCGCACAA |
| *IRE1a* | Human | TGGGTAAAAAGCAGGACATCTGG | GCATAGTCAAAGTAGGTGGCATTCC |
| *VERSICAN* | Human | GAAGGCTTGTTTGGACGTTGG | ACGGAATCCATAAGTCCTGACTC |
| *XBP1* | Human | TTACGAGAGAAAACTCATGGCC | GGGTCCAAGTTGTCCAGAATGC |
| *18S rRNA* | Human | CTCAACACGGGAAACCTCAC | CGCTCCACCAACTAAGAACG |
| *18S rRNA* | Mouse | CTCAACGGAAACCTCAC | AGACAAATCGCTCCACCAAC |

**Supplementary Table 2: List of Antibodies**

| Antibody | Manufacturer | Cat No | Host | | Dilution* |
| --- | --- | --- | --- | --- | --- |
| Beta-actin | Sigma | A2228 | Mouse | | 1:5000 |
| COXIV | Cell Signaling Technology | 4850 | Rabbit | | 1:1000 |
| GAPDH | Cell Signaling Technology | 14C10 | Rabbit | | 1:2000 |
| GCK | Santa Cruz Biotechnology | sc-17819 | Mouse | | 1:1000 |
| Glut 2 | Novus Biologicals | NBP2-22218 | Rabbit | | 1:1000 |
| Glut 4 | Cell Signaling Technology | 2213 | Mouse | | 1:1000 |
| Histone H3 | Cell Signaling Technology | 4499 | Rabbit | | 1:1000 |
| HK1 | Cell Signaling Technology | 2024 | Rabbit | | 1:1000 |
| HK2 | Cell Signaling Technology | 2867 | Rabbit | | 1:1000 |
| HKDC1 | Abcam | ab228729 | Rabbit | | 1:1000 |
| VDAC | Cell Signaling Technology | 4661 | Rabbit | | 1:1000 or 1:200 |
| Geminin | Cell Signaling Technology | 52508 | Rabbit | | 1:1000 |
| PCNA | Cell Signaling Technology | 13110 | Rabbit | | 1:1000 |
| Cyclin D1 | Cell Signaling Technology | 55506 | Rabbit | 1:1000 | |
| Anti-Rabbit | Cell Signaling Technology | 7074 | Goat | 1:10000 | |
| Anti-Mouse | Cell Signaling Technology | 7076 | Horse | 1:10000 | |
| *All dilutions were made in phosphate buffered saline with 5% BSA and 0.1% Tween 20 | | | | | |

**Supplementary Table 3: TCGA study abbreviations**

| ACC | Adrenocortical carcinoma |
| --- | --- |
| BLCA | Bladder Urothelial Carcinoma |
| LGG | Brain Lower Grade Glioma |
| BRCA | Breast invasive carcinoma |
| CESC | Cervical squamous cell carcinoma and endocervical adenocarcinoma |
| CHOL | Cholangiocarcinoma |
| LCML | Chronic Myelogenous Leukemia |
| COAD | Colon adenocarcinoma |
| CNTL | Controls |
| ESCA | Esophageal carcinoma |
| FPPP | FFPE Pilot Phase II |
| GBM | Glioblastoma multiforme |
| HNSC | Head and Neck squamous cell carcinoma |
| KICH | Kidney Chromophobe |
| KIRC | Kidney renal clear cell carcinoma |
| KIRP | Kidney renal papillary cell carcinoma |
| LIHC | Liver hepatocellular carcinoma |
| LUAD | Lung adenocarcinoma |
| LUSC | Lung squamous cell carcinoma |
| DLBC | Lymphoid Neoplasm Diffuse Large B-cell Lymphoma |
| MESO | Mesothelioma |
| MISC | Miscellaneous |
| OV | Ovarian serous cystadenocarcinoma |
| PAAD | Pancreatic adenocarcinoma |
| PCPG | Pheochromocytoma and Paraganglioma |
| PRAD | Prostate adenocarcinoma |
| READ | Rectum adenocarcinoma |
| SARC | Sarcoma |
| SKCM | Skin Cutaneous Melanoma |
| STAD | Stomach adenocarcinoma |
| TGCT | Testicular Germ Cell Tumors |
| THYM | Thymoma |
| THCA | Thyroid carcinoma |
| UCS | Uterine Carcinosarcoma |
| UCEC | Uterine Corpus Endometrial Carcinoma |
| UVM | Uveal Melanoma |
