# Supplementary material for: The hexokinase “HKDC1” interaction with the mitochondria is essential for liver cancer progression
Source: Cell Death Dis. 2022 Jul 28;13(7):660. doi: 10.1038/s41419-022-04999-z (PMC9334634; doi:10.1038/s41419-022-04999-z)

Supplementary Fig 1

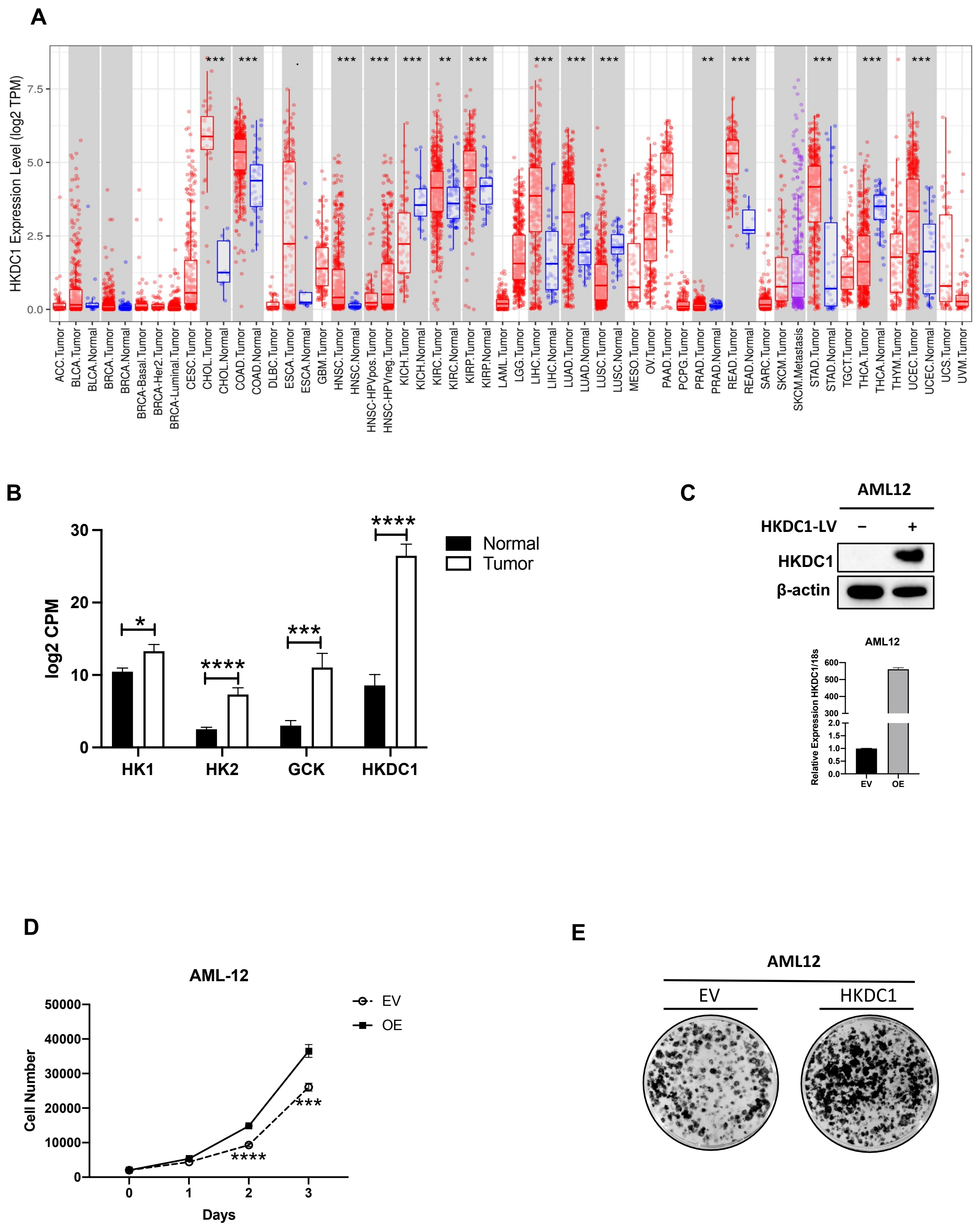

Supplementary Fig 2

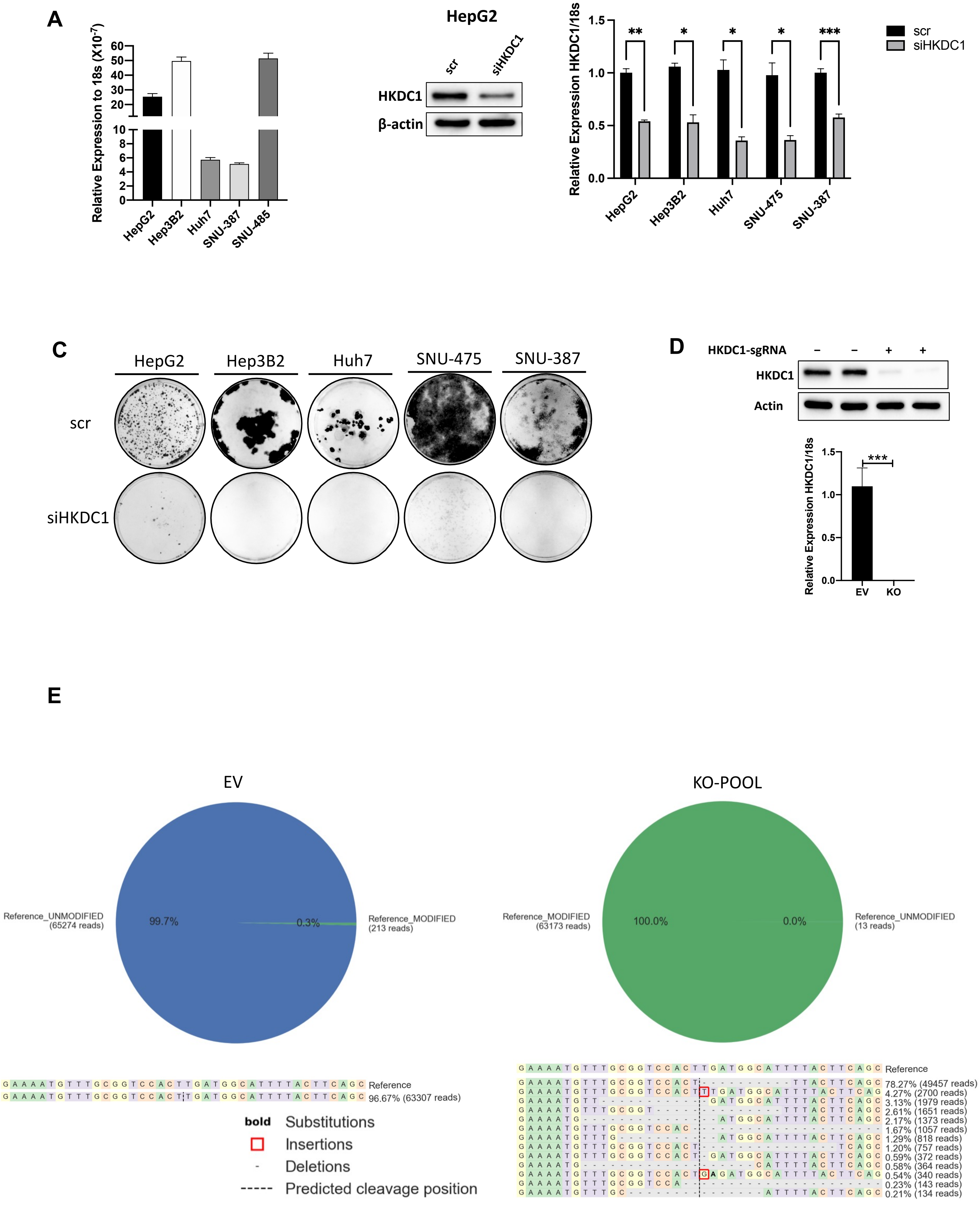

F

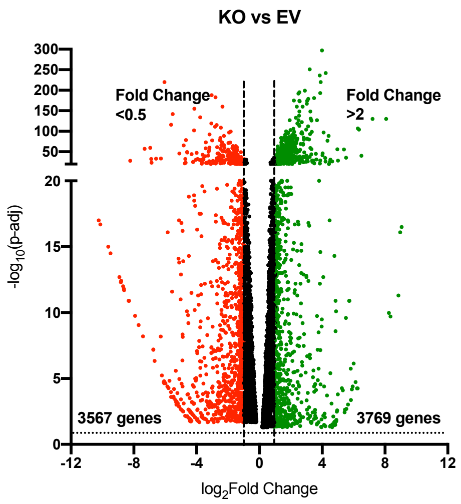
KO vs EV
Fold Change <0.5
Fold Change >2
3567 genes
3769 genes
-log10(p-adj)
log2Fold Change
300
250
200
150
100
50
20
15
10
5
0
-12
-8
-4
0
4
8
12

G

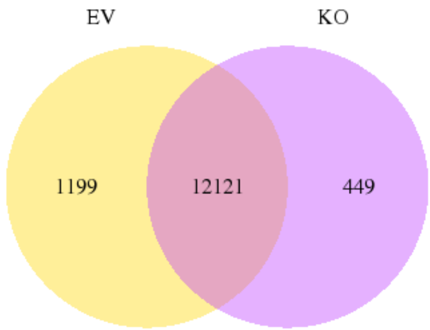
EV
KO
1199
12121
449

H

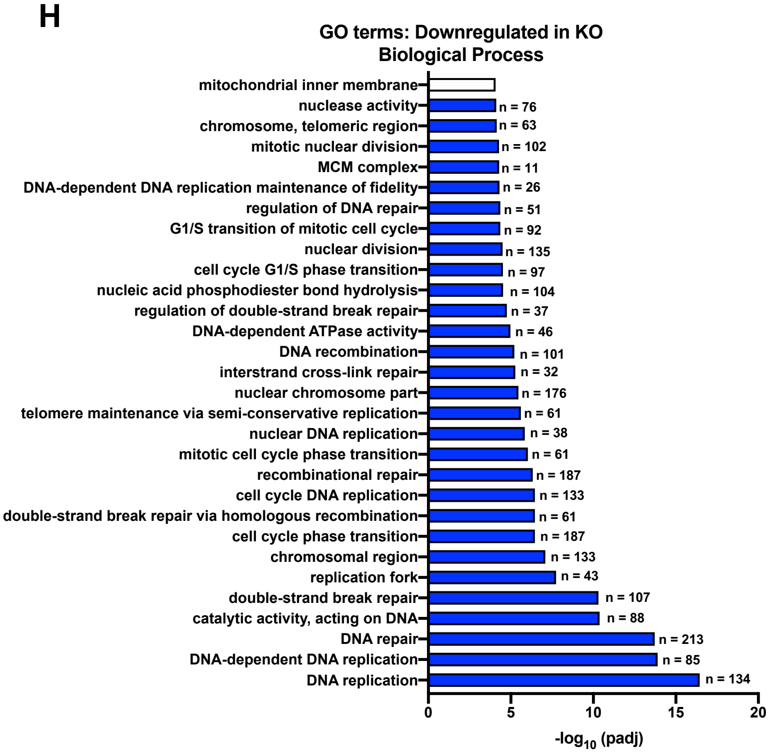
GO terms: Downregulated in KO
Biological Process
mitochondrial inner membrane
nuclease activity
n = 76
chromosome, telomeric region
n = 63
mitotic nuclear division
n = 102
MCM complex
n = 11
DNA-dependent DNA replication maintenance of fidelity
n = 26
regulation of DNA repair
n = 51
G1/S transition of mitotic cell cycle
n = 92
nuclear division
n = 135
cell cycle G1/S phase transition
n = 97
nucleic acid phosphodiester bond hydrolysis
n = 104
regulation of double-strand break repair
n = 37
DNA-dependent ATPase activity
n = 46
DNA recombination
n = 101
interstrand cross-link repair
n = 32
nuclear chromosome part
n = 176
telomere maintenance via semi-conservative replication
n = 61
nuclear DNA replication
n = 38
mitotic cell cycle phase transition
n = 61
recombinational repair
n = 187
cell cycle DNA replication
n = 133
double-strand break repair via homologous recombination
n = 61
cell cycle phase transition
n = 187
chromosomal region
n = 133
replication fork
n = 43
double-strand break repair
n = 107
catalytic activity, acting on DNA
n = 88
DNA repair
n = 213
DNA-dependent DNA replication
n = 85
DNA replication
n = 134
0
5
10
15
20
-log10 (padj)

I

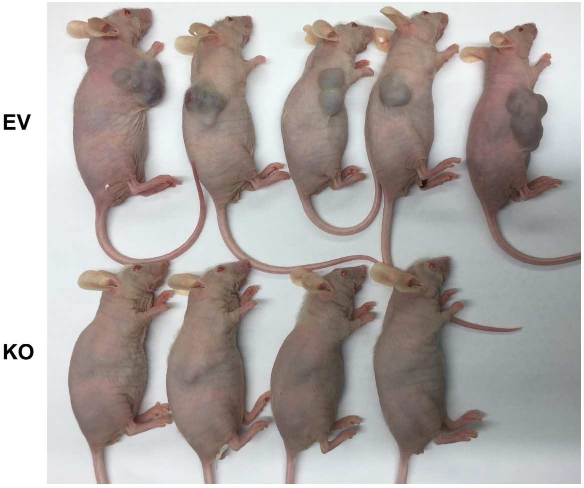
EV
KO

J

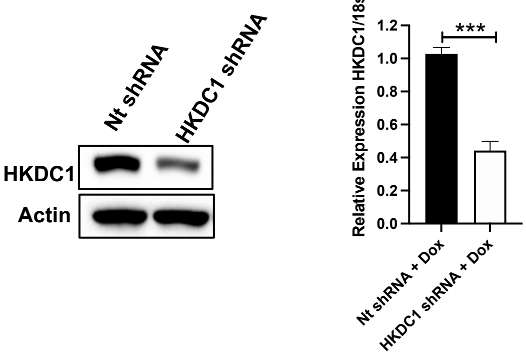
Nt shRNA
HKDC1 shRNA
HKDC1
Actin
Relative Expression HKDC1/18s
1.2
1.0
0.8
0.6
0.4
0.2
0.0
***
Nt shRNA + Dox
HKDC1 shRNA + Dox

K

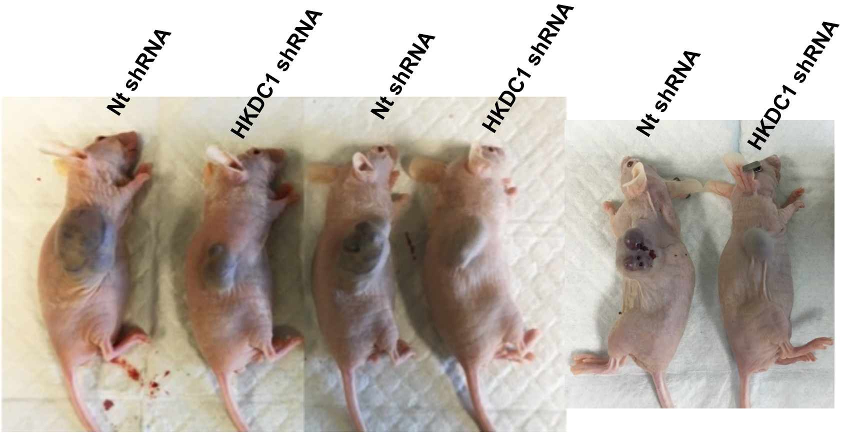
Nt shRNA
HKDC1 shRNA
Nt shRNA
HKDC1 shRNA
Nt shRNA
HKDC1 shRNA

Supplementary Fig 3

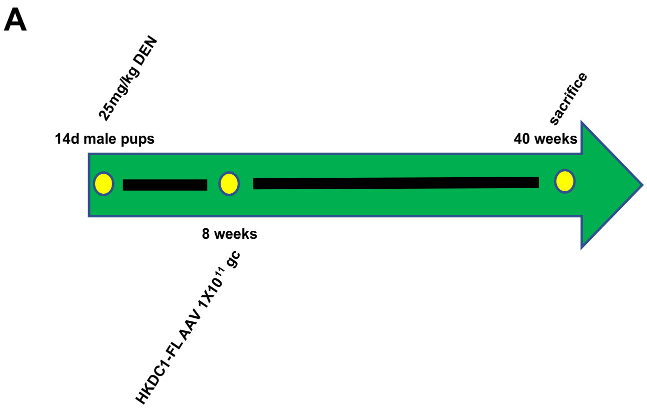

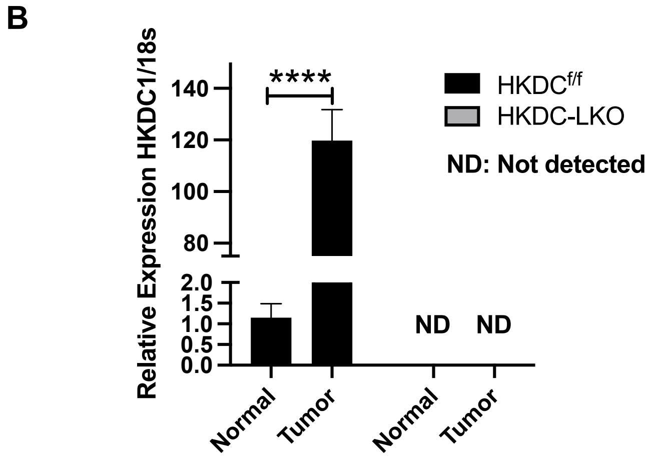

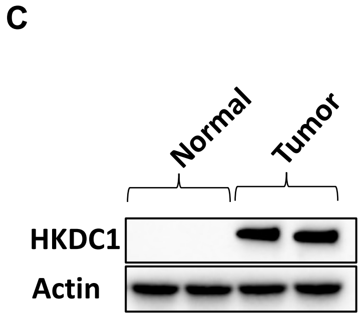

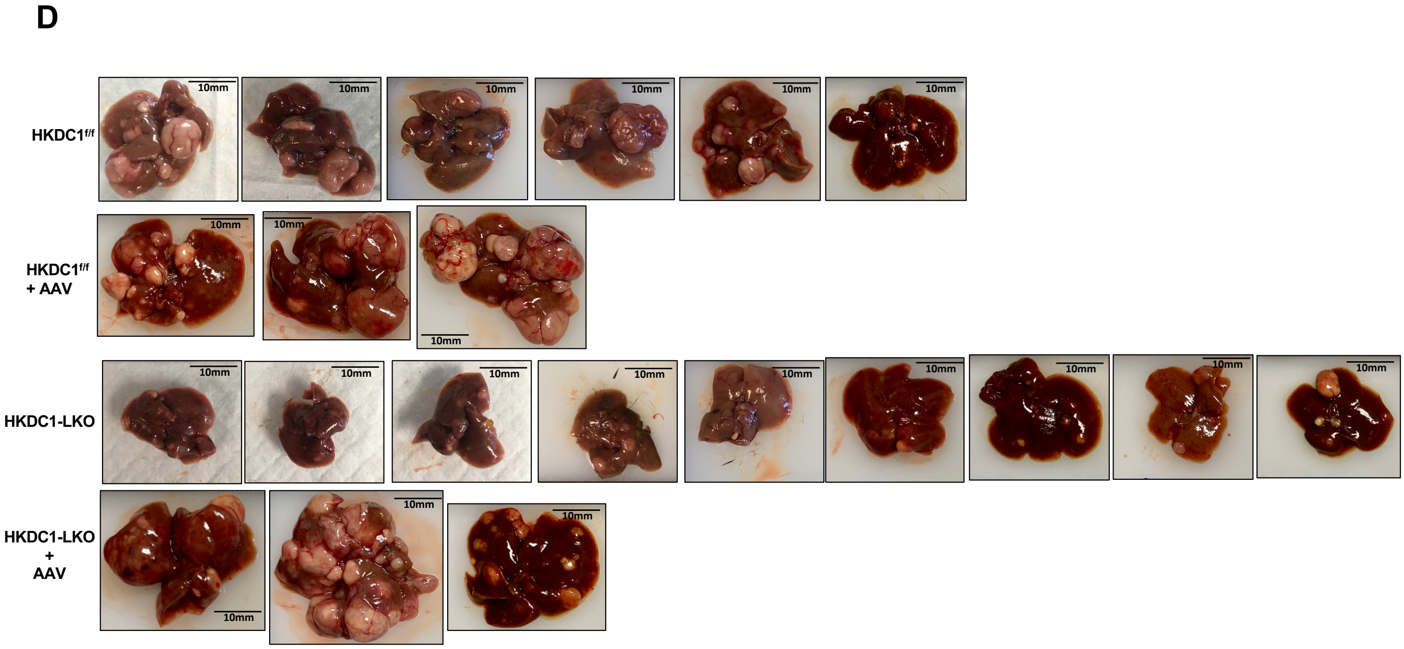

Supplementary Fig 4

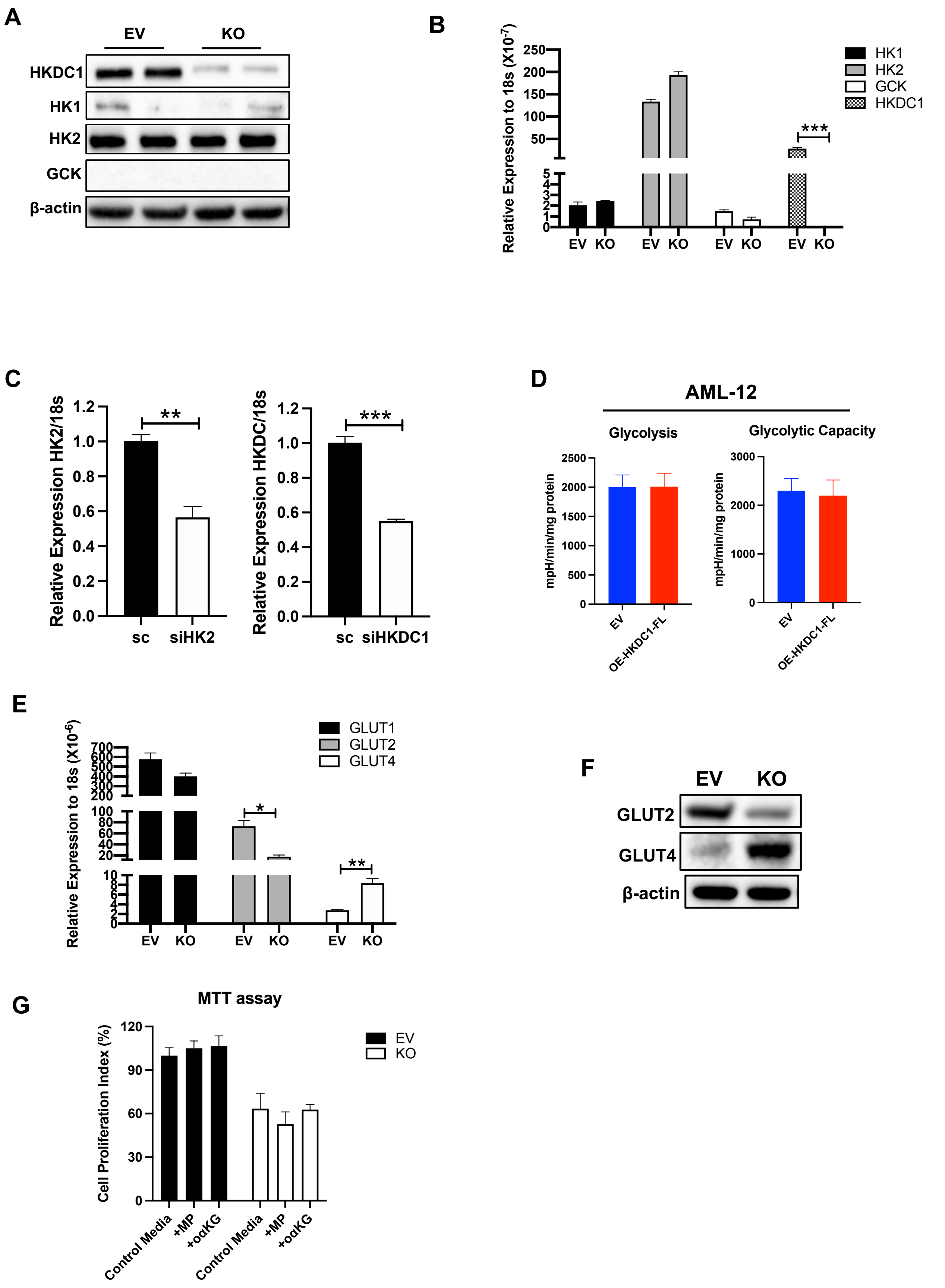

Supplementary Fig 5

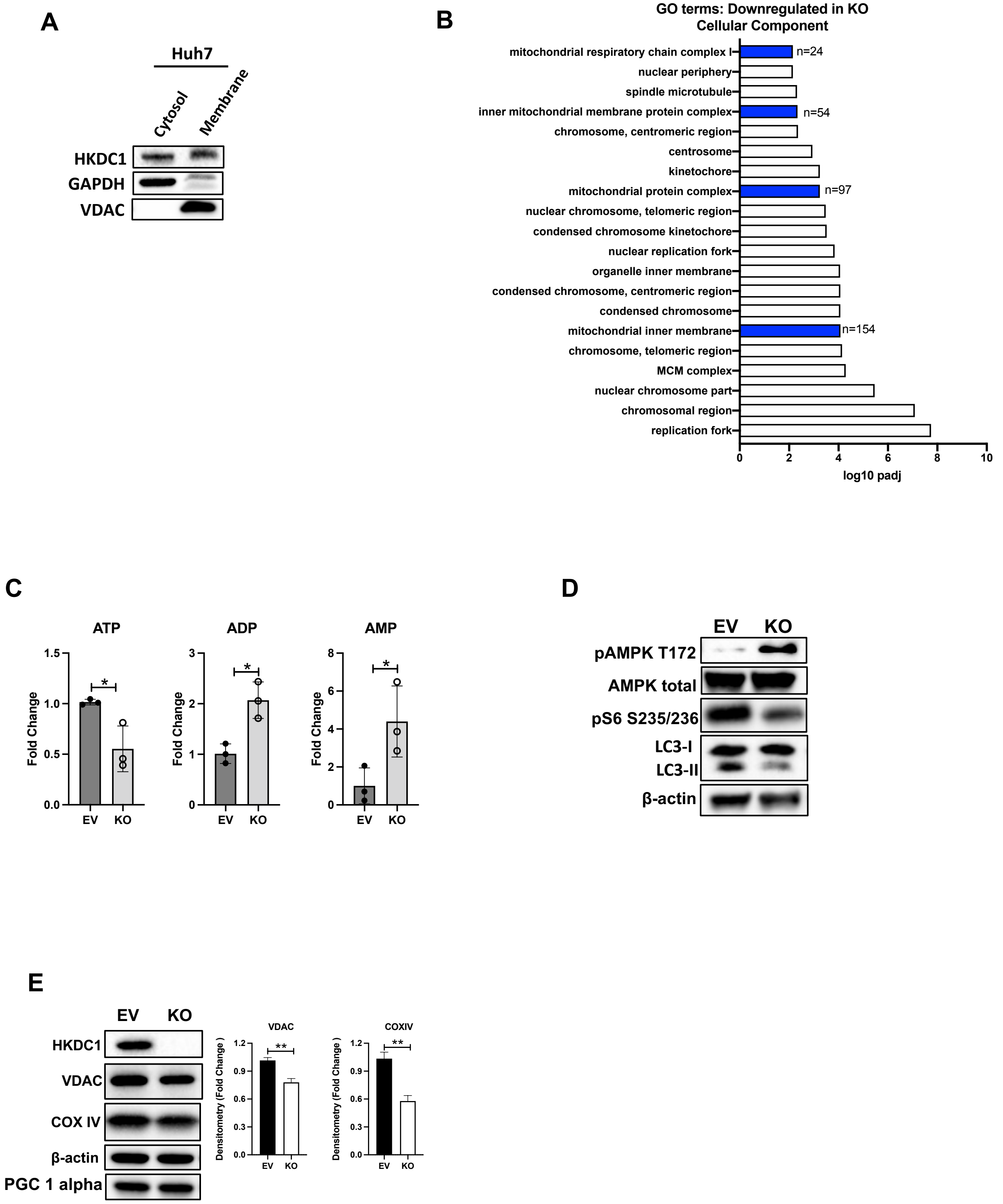

F

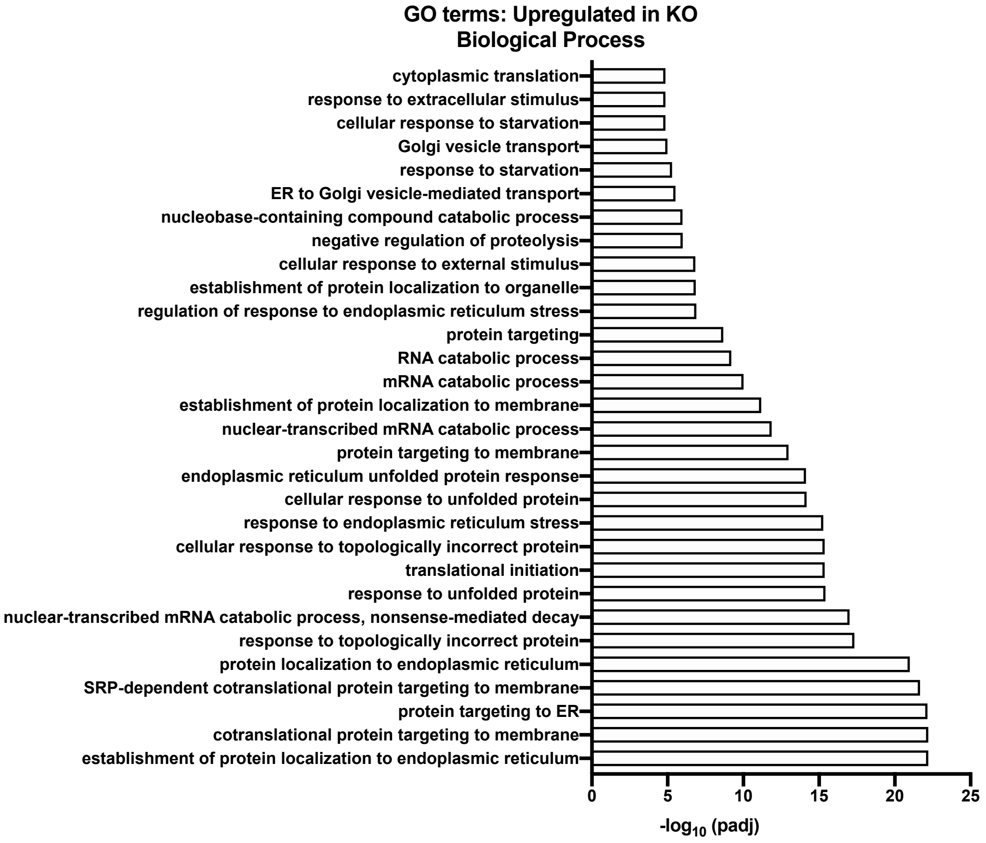
GO terms: Upregulated in KO
Biological Process
cytoplasmic translation
response to extracellular stimulus
cellular response to starvation
Golgi vesicle transport
response to starvation
ER to Golgi vesicle-mediated transport
nucleobase-containing compound catabolic process
negative regulation of proteolysis
cellular response to external stimulus
establishment of protein localization to organelle
regulation of response to endoplasmic reticulum stress
protein targeting
RNA catabolic process
mRNA catabolic process
establishment of protein localization to membrane
nuclear-transcribed mRNA catabolic process
protein targeting to membrane
endoplasmic reticulum unfolded protein response
cellular response to unfolded protein
response to endoplasmic reticulum stress
cellular response to topologically incorrect protein
translational initiation
response to unfolded protein
nuclear-transcribed mRNA catabolic process, nonsense-mediated decay
response to topologically incorrect protein
protein localization to endoplasmic reticulum
SRP-dependent cotranslational protein targeting to membrane
protein targeting to ER
cotranslational protein targeting to membrane
establishment of protein localization to endoplasmic reticulum
0
5
10
15
20
25
-log10 (padj)

Supplementary Fig 6

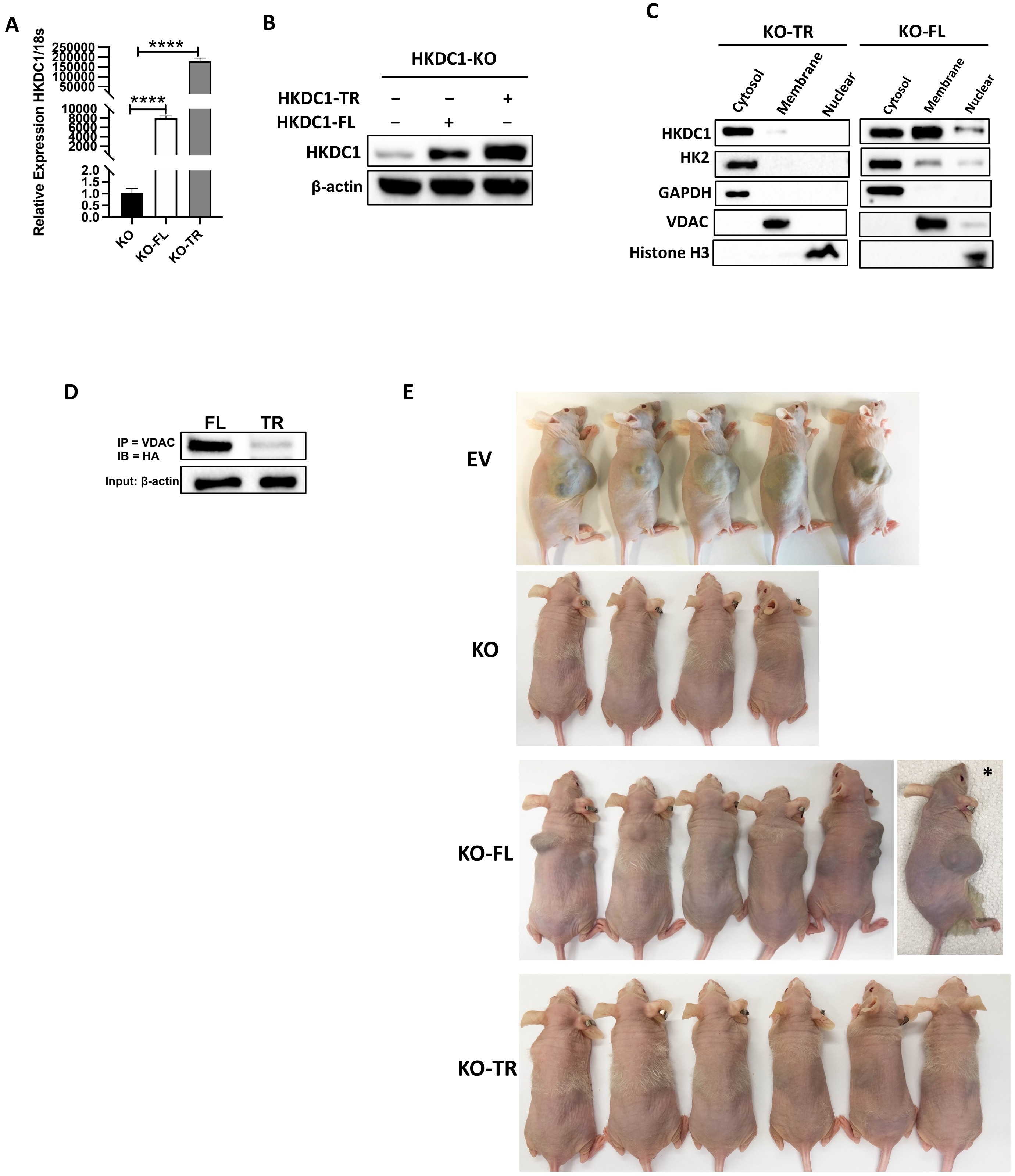

Supplement: Supplementary file 2 — Supplementary Figures [file 41419_2022_4999_MOESM2_ESM.pdf]
