# Supplementary material for: The hexokinase “HKDC1” interaction with the mitochondria is essential for liver cancer progression
Source: Cell Death Dis. 2022 Jul 28;13(7):660. doi: 10.1038/s41419-022-04999-z (PMC9334634; doi:10.1038/s41419-022-04999-z)
Supplement: Supplementary file 4 — Original Data File [file 41419_2022_4999_MOESM4_ESM.pptx]

## Slide 1
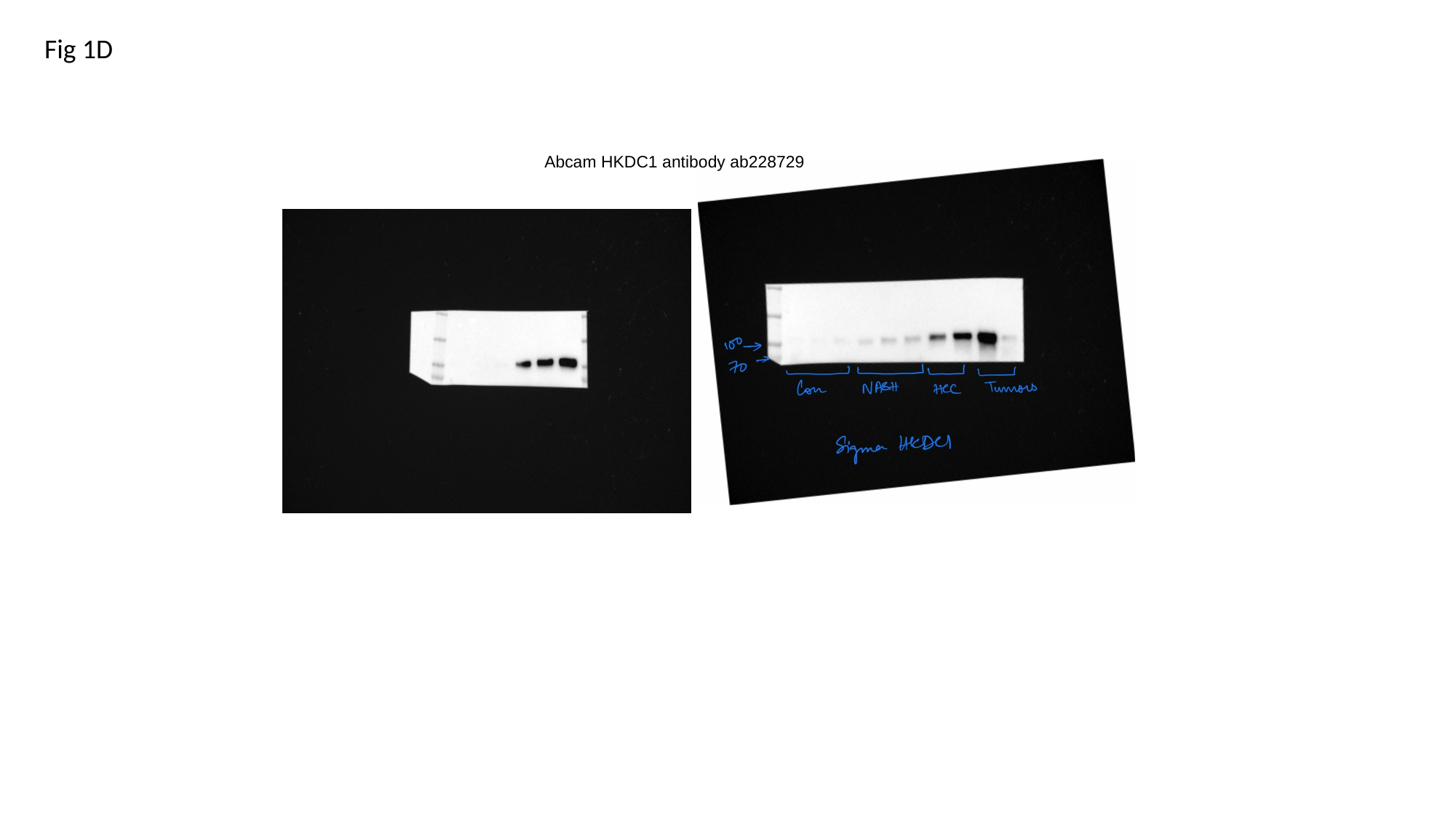

Fig 1D
Abcam HKDC1 antibody ab228729

## Slide 2
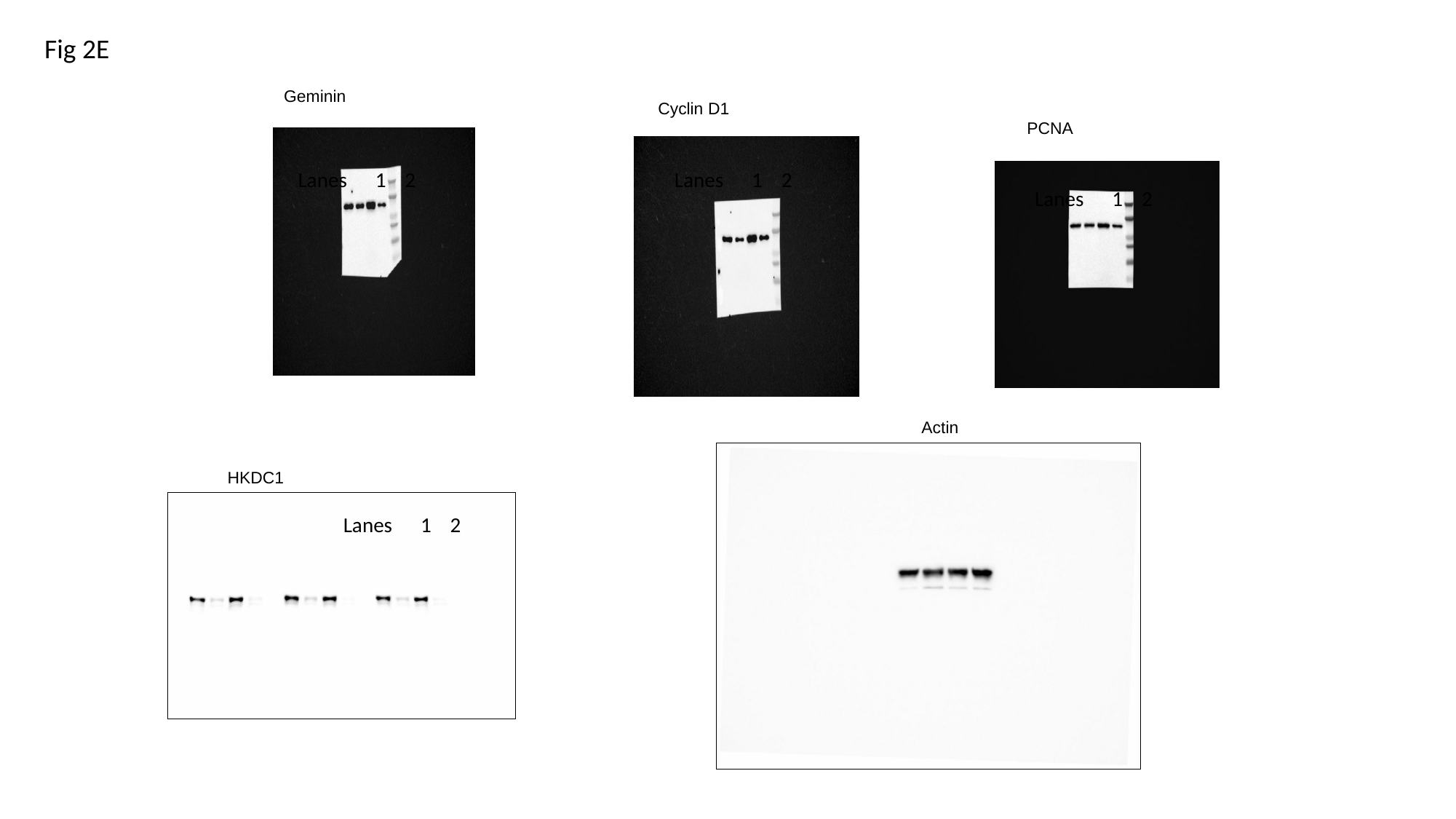

Fig 2E
Geminin
Cyclin D1
PCNA
Lanes 1 2
Lanes 1 2
Lanes 1 2
Actin
HKDC1
Lanes 1 2

## Slide 3
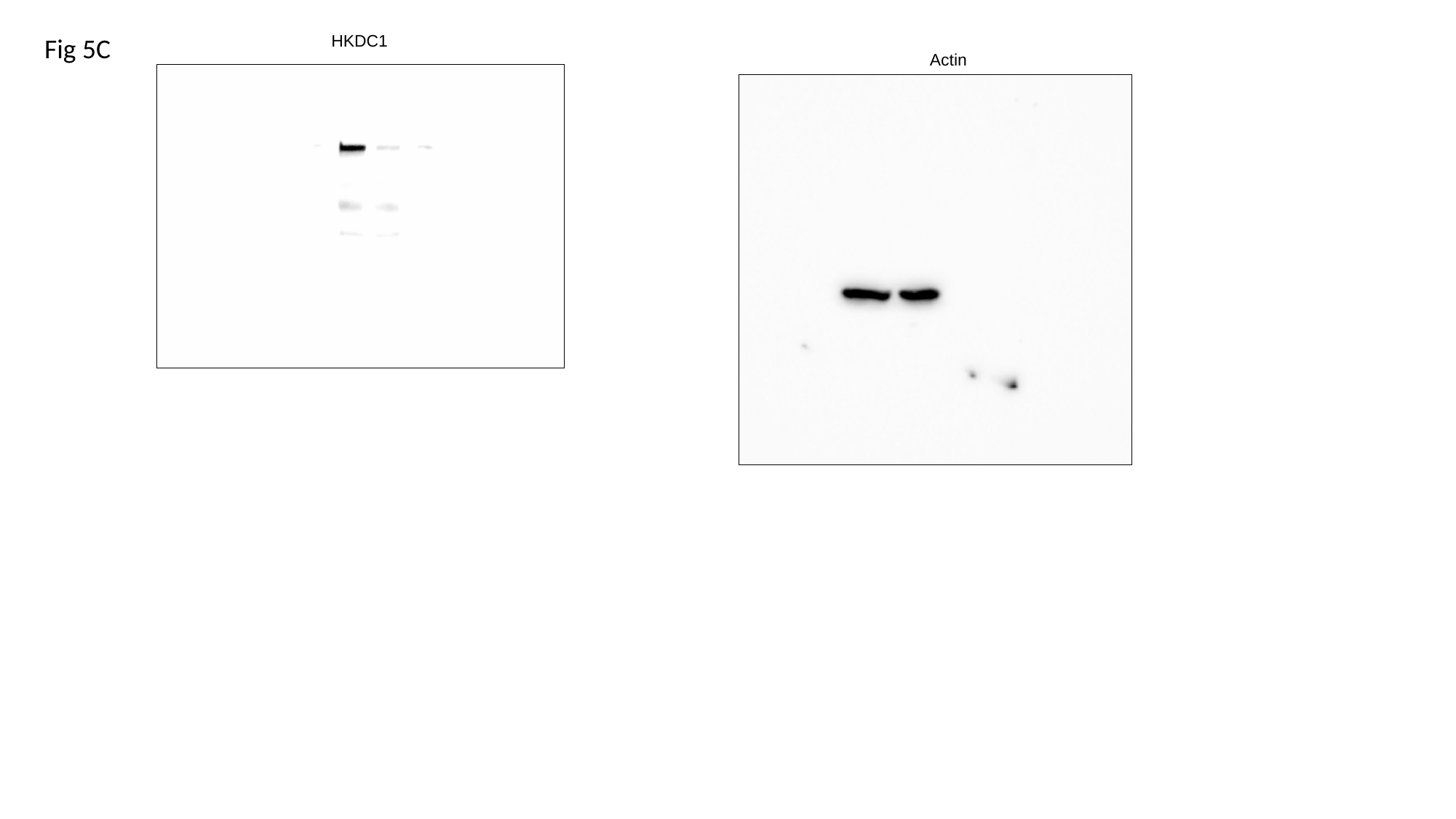

HKDC1
Fig 5C
Actin

## Slide 4
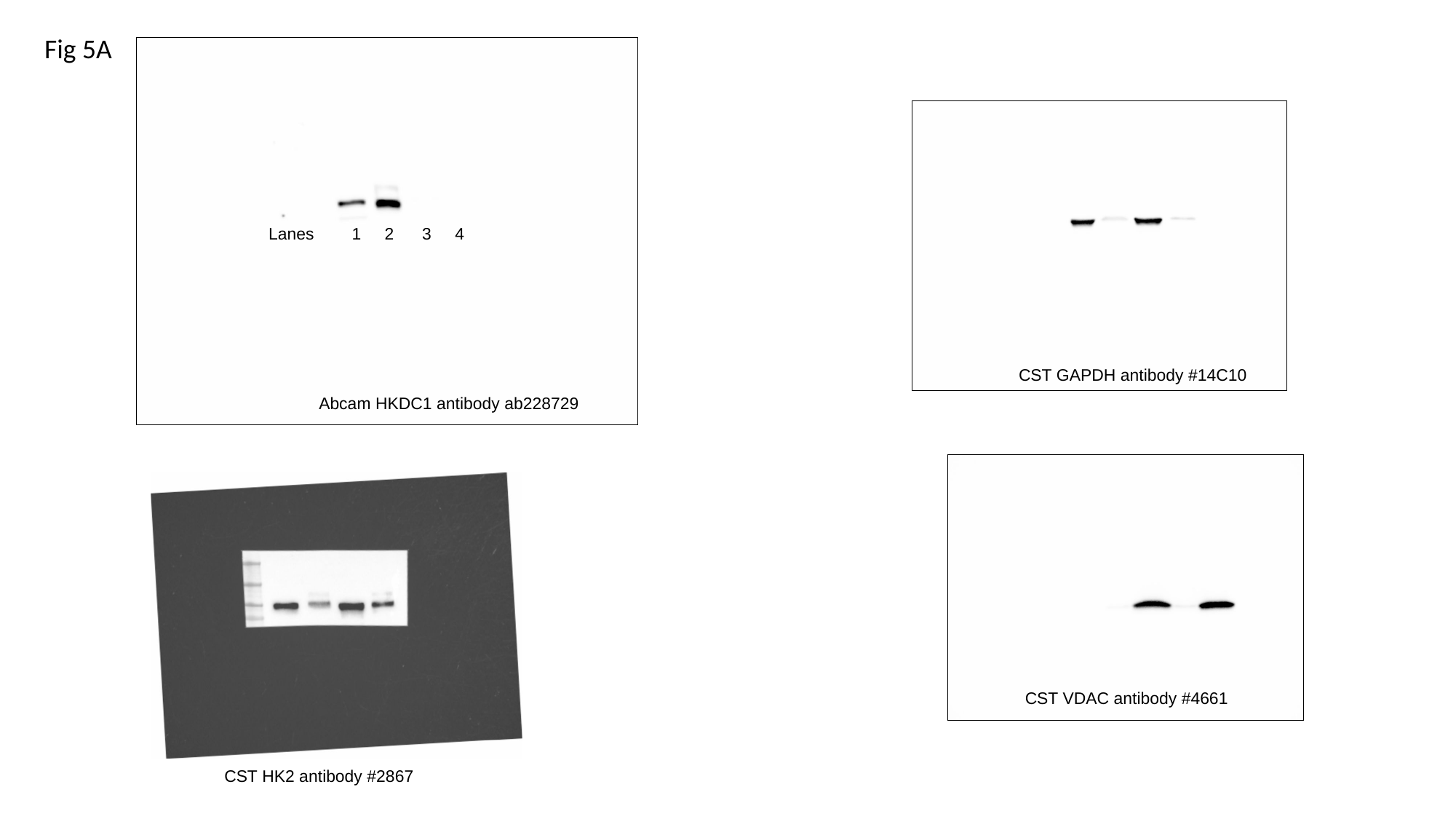

Fig 5A
Lanes 1 2 3 4
CST GAPDH antibody #14C10
Abcam HKDC1 antibody ab228729
CST HK2 antibody #2867
CST VDAC antibody #4661

## Slide 5
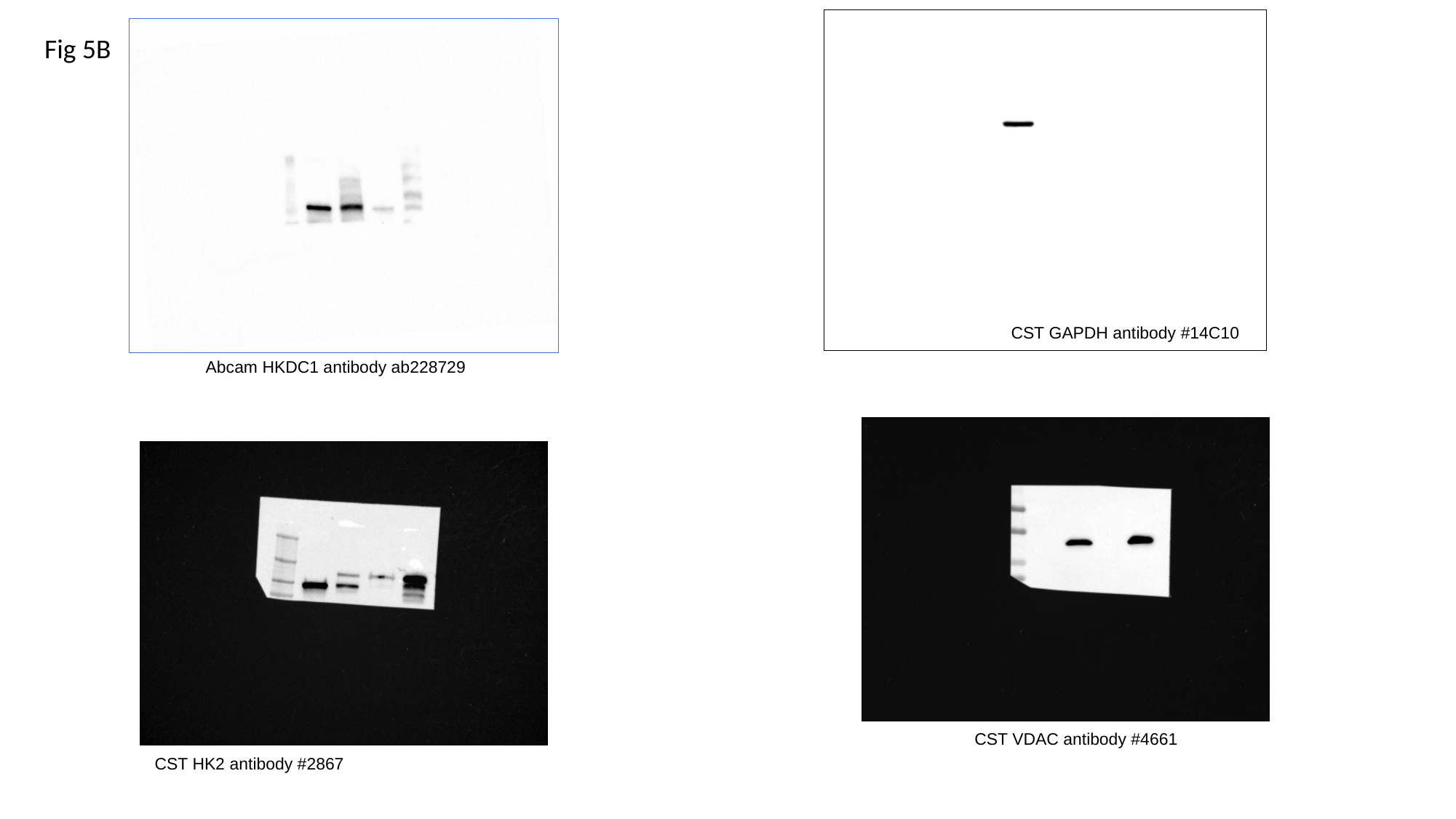

Fig 5B
CST GAPDH antibody #14C10
Abcam HKDC1 antibody ab228729
CST VDAC antibody #4661
CST HK2 antibody #2867
