# Supplementary material for: The hexokinase “HKDC1” interaction with the mitochondria is essential for liver cancer progression
Source: Cell Death Dis. 2022 Jul 28;13(7):660. doi: 10.1038/s41419-022-04999-z (PMC9334634; doi:10.1038/s41419-022-04999-z)
Supplement: Supplementary file 6 — New Co-author addition agreement [file 41419_2022_4999_MOESM6_ESM.pdf]

RE: Our Cell Death & Disease manuscript

From: **mkhan268@uic.edu** Tuesday, May 31, 10:17 AM

To: **Issam Ben-Sahra** | issam.ben-sahra@northwestern.edu, **Alexander Terry** | aterry7@uic.edu, **Priyadarshini, Medha** | mpriya2@uic.edu, **Barton Wicksteed** | bartonw@uic.edu, **MD Wasim Khan** | mkhan268@uic.edu, **Grace Guzman** | graceguz@uic.edu, **Zeenat Farooq** | zeenatfa@uic.edu, **Vladimir Ilievski** | ilievski@uic.edu, **Jose Cordoba** | jcordoba@uic.edu

Cc: **Brian T Layden** | blayde1@uic.edu

Hello everyone,

Our manuscript submitted to Cell Death & Disease has been accepted "In Principle". In our first revision of this manuscript we added the following person as co-authors:

1. Vladimir Ilievski, and
2. Zeenat Farooq

The journal requires that all co-authors are aware and that they accept this. In order to do so please reply to this email indicating whether you accept the addition of these new co-authors. The manuscript revision cannot be submitted unless a response is received from all co-authors.

Thank you

Peace be upon all of us

Md. Wasim Khan, PhD

Assistant Professor of Medicine,

Division of Endocrinology, Diabetes & Metabolism,

Department of Medicine,

M: (312)684-1117

835 S Wolcott Ave, CMWT, Lab 602

Office (Room 808), Chicago, IL 60612

DEPARTMENT OF MEDICINE COLLEGE OF MEDICINE UIC

UI Health CANCER CENTER UIC

From: **mkhan268@uic.edu** Tuesday, May 31, 10:18 AM

To: **Issam Ben-Sahra** | issam.ben-sahra@northwestern.edu, **Alexander Terry** | aterry7@uic.edu, **Priyadarshini, Medha** | mpriya2@uic.edu, **Barton Wicksteed** | bartonw@uic.edu, **Grace Guzman** | graceguz@uic.edu, **Zeenat Farooq** | zeenatfa@uic.edu, **Vladimir Ilievski** | ilievski@uic.edu, **Jose Cordoba** | jcordoba@uic.edu

Cc: **Brian T Layden** | blayde1@uic.edu

I accept the addition of new co-authors.
Wasim Khan

Peace be upon all of us

Md. Wasim Khan, PhD

Assistant Professor of Medicine,
Division of Endocrinology, Diabetes & Metabolism,
Department of Medicine,
M: (312)684-1117
835 S Wolcott Ave, CMWT, Lab 602
Office (Room 808), Chicago, IL 60612

DEPARTMENT OF MEDICINE
COLLEGE OF MEDICINE
UIC

UI Health CANCER CENTER | UIC

From: **Ilievski, Vladimir** | ilievski@uic.edu Tuesday, May 31, 10:20 AM

To: **Khan, Md Wasim** | mkhan268@uic.edu, **Issam Ben-Sahra** | issam.ben-sahra@northwestern.edu, **Terry, Alexander Richard** | aterry7@uic.edu, **Priyadarshini, Medha** | mpriya2@uic.edu, **Wicksteed, Barton L** | bartonw@uic.edu, **Guzman, Grace** | GraceGuz@uic.edu, **Farooq, Zeenat** | zeenatfa@uic.edu, **Cordoba-Chacon, Jose** | jcordoba@uic.edu

Cc: **Layden, Brian** | blayde1@uic.edu

I accept the addition of new co-authors.
Vladimir Ilievski

Sent from [Mail](#) for Windows

From: **Priyadarshini, Medha** | mpriya2@uic.edu — Tuesday, May 31, 10:22 AM

To: **Khan, Md Wasim** | mkhan268@uic.edu, **Issam Ben-Sahra** | issam.ben-sahra@northwestern.edu, **Terry, Alexander Richard** | aterry7@uic.edu, **Wicksteed, Barton L** | bartonw@uic.edu, **Guzman, Grace** | GraceGuz@uic.edu, **Farooq, Zeenat** | zeenatfa@uic.edu, **Ilievski, Vladimir** | ilievski@uic.edu, **Cordoba-Chacon, Jose** | jcordoba@uic.edu

Cc: **Layden, Brian** | blayde1@uic.edu

I accept the addition of new co-authors.

Best regards,

Medha

Medha Priyadarshini, Ph.D.
Research Assistant Professor
Department of Medicine
Division of Endocrinology, Diabetes & Metabolism
University of Illinois at Chicago

I alone cannot change the world, but I can cast a stone across the waters to create many ripples.

From: **Cordoba-Chacon, Jose** | jcordoba@uic.edu — Tuesday, May 31, 10:23 AM

To: **Khan, Md Wasim** | mkhan268@uic.edu, **Issam Ben-Sahra** | issam.ben-sahra@northwestern.edu, **Terry, Alexander Richard** | aterry7@uic.edu, **Priyadarshini, Medha** | mpriya2@uic.edu, **Wicksteed, Barton L** | bartonw@uic.edu, **Guzman, Grace** | GraceGuz@uic.edu, **Farooq, Zeenat** | zeenatfa@uic.edu, **Ilievski, Vladimir** | ilievski@uic.edu

Cc: **Layden, Brian** | blayde1@uic.edu

I accept the addition of new co-authors.

Please send the submitted version.

Thanks
Jose

From: **Issam Ben-Sahra** | issam.ben-sahra@northwestern.edu Tuesday, May 31, 11:45 AM

To: **Cordoba-Chacon, Jose** | jcordoba@uic.edu

Cc: **mkhan268** | mkhan268@uic.edu, **Terry, Alexander Richard** | aterry7@uic.edu, **Priyadarshini, Medha** | mpriya2@uic.edu, **Wicksteed, Barton L** | bartonw@uic.edu, **Guzman, Grace** | GraceGuz@uic.edu, **Farooq, Zeenat** | zeenatfa@uic.edu, **Ilievski, Vladimir** | ilievski@uic.edu, **blayde1** | blayde1@uic.edu

I accept the addition of new co-authors.

Thank you,

Issam

From: **Alexander R Terry** | aterry7@uic.edu Tuesday, May 31, 12:22 PM

To: **Md Wasim Khan** | mkhan268@uic.edu

I accept addition of new co-authors.

Best,
Alex

From: **Wicksteed, Barton L** | bartonw@uic.edu Tuesday, May 31, 12:50 PM

To: **Khan, Md Wasim** | mkhan268@uic.edu

I accept the addition of the new authors
Barton Wicksteed

From: **Brian Layden** | blayde1@uic.edu Tuesday, May 31, 1:31 PM

To: **Ilievski, Vladimir** | ilievski@uic.edu

Cc: **Khan, Md Wasim** | mkhan268@uic.edu, **Issam Ben-Sahra** | issam.ben-sahra@northwestern.edu, **Terry, Alexander Richard** | aterry7@uic.edu, **Priyadarshini, Medha** | mpriya2@uic.edu, **Wicksteed, Barton L** | bartonw@uic.edu, **Guzman, Grace** | GraceGuz@uic.edu, **Farooq, Zeenat** | zeenatfa@uic.edu, **Cordoba-Chacon, Jose** | jcordoba@uic.edu

I accept the addition of new co-authors.

--
Brian T. Layden, M.D., Ph.D.
Associate Professor

Chief, Division of Endocrinology, Diabetes and Metabolism
University of Illinois at Chicago

*********************EMAIL DISCLAIMER********************* This email and any files transmitted with it may be confidential and are intended solely for the use of the individual or entity to whom they are addressed. If you are not the intended recipient or the individual responsible for delivering the e-mail to the intended recipient, any disclosure, copying, distribution or any action taken or omitted to be taken in reliance on it, is strictly prohibited. If you have received this e-mail in error, please delete it and notify the sender or contact Privacy Office 312.355.5650.

From: **Farooq, Zeenat** | zeenatfa@uic.edu — Tuesday, May 31, 4:50 PM

To: **Ilievski, Vladimir** | ilievski@uic.edu, **Khan, Md Wasim** | mkhan268@uic.edu, **Issam Ben-Sahra** | issam.ben-sahra@northwestern.edu, **Terry, Alexander Richard** | aterry7@uic.edu, **Priyadarshini, Medha** | mpriya2@uic.edu, **Wicksteed, Barton L** | bartonw@uic.edu, **Guzman, Grace** | GraceGuz@uic.edu, **Cordoba-Chacon, Jose** | jcordoba@uic.edu

Cc: **Layden, Brian** | blayde1@uic.edu

I accept the addition of new co-authors.

Zeenat Farooq

From: **Guzman, Grace** | GraceGuz@uic.edu — Tuesday, May 31, 8:12 PM

To: **Farooq, Zeenat** | zeenatfa@uic.edu, **Ilievski, Vladimir** | ilievski@uic.edu, **Khan, Md Wasim** | mkhan268@uic.edu, **Issam Ben-Sahra** | issam.ben-sahra@northwestern.edu, **Terry, Alexander Richard** | aterry7@uic.edu, **Priyadarshini, Medha** | mpriya2@uic.edu, **Wicksteed, Barton L** | bartonw@uic.edu, **Cordoba-Chacon, Jose** | jcordoba@uic.edu

Cc: **Layden, Brian** | blayde1@uic.edu

I accept the addition of new co-authors.

Grace

Grace Guzman, MD

Associate Professor

Pathology

University of Illinois at Chicago

College of Medicine

UI Health

GraceGuz@uic.edu

Office: 312 996-3886

840 South Wood Street

Room 130 60612 MC 847
