# Supplementary material for: The hexokinase “HKDC1” interaction with the mitochondria is essential for liver cancer progression
Source: Cell Death Dis. 2022 Jul 28;13(7):660. doi: 10.1038/s41419-022-04999-z (PMC9334634; doi:10.1038/s41419-022-04999-z)
Supplement: Supplementary file 7 — Author Contribution Form [file 41419_2022_4999_MOESM7_ESM.pdf]

DECLARATION OF CONTRIBUTIONS TO ARTICLE

ADMC

Manuscript Number: CDD-21-2786

Journal Name: *Cell Death & Differentiation* (the 'Journal')

Proposed Title of the Contribution:

The hexokinase "HKDC1" interaction with the mitochondria is essential for liver (the 'Contribution)

Author(s):

MWK, ART, MP, GG, JCC, IBS, BW, BTL (the 'Authors')

For all *CDD* articles, each person named as an author in the published version must be able to show he or she has contributed substantially to the article.

Authorship credit should be based on 1) substantial contributions to conception and design, acquisition of data, or analysis and interpretation of data; 2) drafting the article or revising it critically for important intellectual content; and 3) final approval of the version to be published. Authors should meet conditions 1, 2 and 3.

Any person who cannot be shown to have made a substantial contribution to the article cannot be listed as an author in the final version. The name of any person who is deemed to have made a minor contribution can, however, appear in the Acknowledgments section of the article.

Please complete the table below to indicate the contributions of all named authors to the manuscript.

Author Full Name:	Specification of Contribution to the Manuscript:
Md. Wasim Khan	designed and performed experiments, analyzed, and interpreted the results, and wrote the manuscript
Alexander R. Terry	Seahorse experiments
Medha Priyadarshini	cell proliferation assays
Grace Guzman	provided liver samples for mouse NASH
Jose Cordoba-Chacon	assisted with glucose consumption assay
Issam Ben-Sahra	metabolomics assays
Barton Wicksteed	DEN mouse model
Brian T. Layden	designed the project, analyzed, and interpreted the data, and wrote the manuscript.

Please complete the table below to indicate the contributions of all named authors to the figures.

Figure 1:
MWK, JCC

Figure 2:
MWK, MP

Figure 3:
MWK, GG

Figure 4:
MWK, AT, IBS, BW

Figure 5:
MWK

Figure 6:
MWK

Signed for and on behalf of the Author(s):	Print Name:	Date:
Brian Layden	Brian T Layden	12/13/2021
